# Dynamic conformational changes of a tardigrade group-3 late embryogenesis abundant protein modulate membrane biophysical properties

**DOI:** 10.1093/pnasnexus/pgae006

**Published:** 2024-01-18

**Authors:** Xiao-Han Li, Conny W H Yu, Natalia Gomez-Navarro, Viktoriya Stancheva, Hongni Zhu, Andal Murthy, Michael Wozny, Ketan Malhotra, Christopher M Johnson, Martin Blackledge, Balaji Santhanam, Wei Liu, Jinqing Huang, Stefan M V Freund, Elizabeth A Miller, M Madan Babu

**Affiliations:** MRC Laboratory of Molecular Biology, Cambridge CB2 0QH, UK; MRC Laboratory of Molecular Biology, Cambridge CB2 0QH, UK; MRC Laboratory of Molecular Biology, Cambridge CB2 0QH, UK; MRC Laboratory of Molecular Biology, Cambridge CB2 0QH, UK; Department of Chemistry, The Hong Kong University of Science and Technology, Clear Water Bay, Hong Kong, China; MRC Laboratory of Molecular Biology, Cambridge CB2 0QH, UK; MRC Laboratory of Molecular Biology, Cambridge CB2 0QH, UK; MRC Laboratory of Molecular Biology, Cambridge CB2 0QH, UK; MRC Laboratory of Molecular Biology, Cambridge CB2 0QH, UK; Université Grenoble Alpes, CNRS, Commissariat à l’Energie Atomique et aux Energies Alternatives, Institut de Biologie Structurale, 38000 Grenoble, France; MRC Laboratory of Molecular Biology, Cambridge CB2 0QH, UK; Department of Structural Biology, Center of Excellence for Data-Driven Discovery, St Jude Children's Research Hospital, Memphis, TN 38105, USA; Department of Chemistry, State Key Laboratory of Synthetic Chemistry, The University of Hong Kong, Pokfulam Road, Hong Kong, China; Department of Chemistry, The Hong Kong University of Science and Technology, Clear Water Bay, Hong Kong, China; MRC Laboratory of Molecular Biology, Cambridge CB2 0QH, UK; MRC Laboratory of Molecular Biology, Cambridge CB2 0QH, UK; MRC Laboratory of Molecular Biology, Cambridge CB2 0QH, UK; Department of Structural Biology, Center of Excellence for Data-Driven Discovery, St Jude Children's Research Hospital, Memphis, TN 38105, USA

**Keywords:** intrinsically disordered proteins, conformational dynamics, protein–membrane interactions, late embryogenesis abundant proteins

## Abstract

A number of intrinsically disordered proteins (IDPs) encoded in stress-tolerant organisms, such as tardigrade, can confer fitness advantage and abiotic stress tolerance when heterologously expressed. Tardigrade-specific disordered proteins including the cytosolic-abundant heat-soluble proteins are proposed to confer stress tolerance through vitrification or gelation, whereas evolutionarily conserved IDPs in tardigrades may contribute to stress tolerance through other biophysical mechanisms. In this study, we characterized the mechanism of action of an evolutionarily conserved, tardigrade IDP, HeLEA1, which belongs to the group-3 late embryogenesis abundant (LEA) protein family. HeLEA1 homologs are found across different kingdoms of life. HeLEA1 is intrinsically disordered in solution but shows a propensity for helical structure across its entire sequence. HeLEA1 interacts with negatively charged membranes via dynamic disorder-to-helical transition, mainly driven by electrostatic interactions. Membrane interaction of HeLEA1 is shown to ameliorate excess surface tension and lipid packing defects. HeLEA1 localizes to the mitochondrial matrix when expressed in yeast and interacts with model membranes mimicking inner mitochondrial membrane. Yeast expressing HeLEA1 shows enhanced tolerance to hyperosmotic stress under nonfermentative growth and increased mitochondrial membrane potential. Evolutionary analysis suggests that although HeLEA1 homologs have diverged their sequences to localize to different subcellular organelles, all homologs maintain a weak hydrophobic moment that is characteristic of weak and reversible membrane interaction. We suggest that such dynamic and weak protein–membrane interaction buffering alterations in lipid packing could be a conserved strategy for regulating membrane properties and represent a general biophysical solution for stress tolerance across the domains of life.

**Significance Statement**

Late embryogenesis abundant (LEA) proteins are a large family of intrinsically disordered proteins (IDPs) that can confer abiotic stress tolerance. Despite the extensive phenotypic and biophysical characterization of LEA proteins, how their conserved sequence and structural features drive their function remains to be fully elucidated. We characterized a tardigrade group-3 LEA protein, HeLEA1, using an integrative structural and computational approach to reveal evolutionarily conserved structural and sequence features that contribute to its function. We uncover that dynamic interaction with negatively charged membranes via reversible disorder-to-helical transitions of HeLEA1 allows modulation of the mechanical and thermodynamic properties of lipid bilayers. Our study reveals a synergy between subcellular location, disordered state conformation, and biophysical properties of transient structured states in the evolution of IDPs.

## Introduction

Organisms often face environmental challenges that can affect their survival. Such challenges include extreme temperatures (heat or cold shock), water stress (desiccation or freezing), as well as altered osmotic pressure, and can result in substantial damage to proteins, membranes, and genetic material (1–3). To survive these conditions, organisms adopt multiple strategies. Whereas large animals can physically move from harsh environments, organisms such as plants, microbes, and invertebrates that cannot physically move or move quickly have evolved cellular mechanisms to combat abiotic stress. One common response to diverse stresses is the expression of proteins that can increase levels of intracellular osmolytes and chemical chaperones such as trehalose, betaine, glycine (4, 5), and/or proteins that have a direct protective role on cellular components (6, 7). A subset of the latter category includes proteins with no defined tertiary structure, typically referred to as intrinsically disordered proteins (IDPs) (8).

Tardigrades, or water bears, are organisms known for withstanding extreme environmental conditions, and they have been reported to increase the expression of certain IDPs upon exposure to desiccation (9). Heterologous expression of these tardigrade IDPs in unicellular organisms such as *Saccharomyces cerevisiae* enhances their tolerance to desiccation (9). The desiccation stress-tolerant phenotype has been largely associated with vitrification or gelation of cytosolic-abundant heat-soluble (CAHS) proteins (9–13), whereas other tardigrade IDPs such as late embryogenesis abundant (LEA) proteins or mitochondrial abundant heat-soluble proteins (MAHSs) have been reported to be able to confer tolerance to osmotic stresses when heterologously expressed in mammalian cells (14).

Despite the prevalence of these proteins and extensive functional studies on them, how the sequence and structural features of such disordered proteins translate into their function is less well understood, especially for tardigrade IDPs other than CAHS proteins. Herein, we comprehensively characterized a tardigrade stress-tolerance protein, which had previously been defined as a CAHS protein (9). We show that this protein, which we now name HeLEA1, belongs to the group-3 LEA protein family and undergoes a dynamic disorder-to-helical transition. HeLEA1 interacts with negatively charged lipids and buffers synthetic lipid bilayers from packing defects and excess membrane tension. NMR structural analysis supports that the conserved LEA motifs in HeLEA1 overlap with regions that have higher local disorder propensity at an early stage of disorder-to-helical transition. Sequence analysis reveals that HeLEA1 homologs have diverged in their sequences to different subcellular locations; all homologs maintain a weak hydrophobic moment that is characteristic of weak and reversible protein–membrane interactions. We propose that natural selection has preserved sequence features in LEA proteins that drive both a disordered state and a membrane-bound structured state to maintain the integrity of lipid bilayers during stress.

## Results

### HeLEA1 belongs to a family of group-3 LEA proteins that is intrinsically disordered with predicted helical conformation

**Fig. 1.**
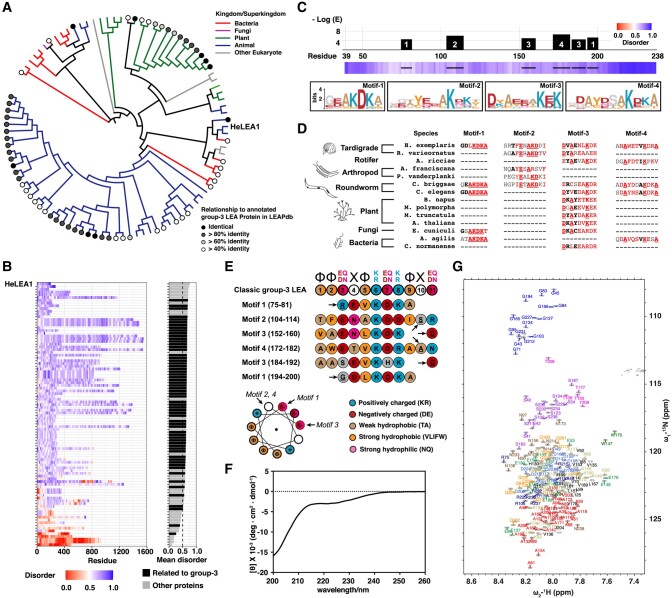
HeLEA1 is an intrinsically disordered group-3 LEA protein. A) Evolutionary tree of HeLEA1 homologs annotated by their host kingdom (branch color) and sequence identity and their relationship to annotated group-3 LEA protein in LEA protein database (LEAPdb, Data S1) (18). B) Residue-specific disorder prediction for 96 HeLEA1 homologs using IUPred2A (left) sorted by the mean disorder scores across their sequences (right). Homologs sharing more than 40% sequence identity with annotated group-3 LEA protein in LEAPdb are represented as the darker bars. Eighty-three of 96 HeLEA1 homologs have a mean IUPred disorder score >0.5. HeLEA1 have the highest mean disorder propensity. C) HeLEA1 possesses multiple LEA protein motifs. Top panel shows residue-specific confidence score of LEA motif in HeLEA1 (*E*-score < 1E–4, MEME algorithm (22)). Bottom panel shows disorder prediction for HeLEA1 using IUPred2A. The locations of each high-confidence motif are indicated by bold lines, and the corresponding motif logos shown below. D) Local motif alignments between HeLEA1 and representative HeLEA1 homologs from diverse species. Residues that share >70% identity in amino acids are highlighted by underline and red color, >80% similarity in amino acid properties are highlighted by red color, >60% similarity in amino acid properties are highlighted in bold and black. E) Comparison of group-3 LEA protein motifs found in HeLEA1 with reported classic group-3 LEA protein motif. Amino acids are colored by their biophysical properties. The start of each motif is marked by an arrow. Less conserved positions on the helical wheel are marked by dashed lines. F) CD spectra of 10 μM HeLEA1 in solution. The spectrum lacks the characteristic signals of alpha-helical (negative peaks at 222 and 208 nm) and beta-sheet (negative peak at 218 nm) secondary structures and has a characteristic signal of a disordered protein (negative peak at 200 nm). G) The ^1^H_N_,^15^N 2D HSQC of HeLEA1 purified from *E. coli* shown with assignment of backbone resonances. Residues are colored according to their amino acid types. Detailed full assignment of the HSQC spectrum is available in Fig. S4.

Previous work identified four genes of the tardigrade species *Hypsibius exemplaris* encoding IDPs that conferred desiccation tolerance when heterologously expressed in *S. cerevisiae* or *Escherichia coli* (9). Additionally, proteomics and comparative transcriptomics of tardigrades have suggested that such IDPs are prevalent in other stress-tolerant tardigrade species (14, 15). We sought to exhaustively identify all remote homologs of these four *H. exemplaris* proteins using a comprehensive sequence search with HMMer (16). We identified 144 homologs of the four protective proteins, subjected them to an all-against-all sequence comparison, and clustered them using the enzyme function initiative similarity tool (17). Our analysis identified two clusters: three of the four queried sequences belong to the CAHS protein family, which contains only tardigrade proteins (Fig. S1 and Table S1; 48 sequences from three tardigrade species). The fourth protein belongs to a different family that is evolutionarily conserved and clusters with annotated group-3 LEA proteins or proteins sharing sequence homology with group-3 LEA proteins from the LEAP database (18) (Pfam: PF02987, group 3 (19), LEA_4 (20), or group 6 (21) according to different classifications, Data S1) (Figs. 1A and S1, Table S2; 96 sequences from 44 species, including two tardigrade species). We renamed this tardigrade protein, which was previously classified as a CAHS protein ((9), UniProtID: P0CU49) as HeLEA1 (i.e. LEA protein from *H. exemplaris*).

LEA proteins are among the earliest IDP families discovered to confer protection against multiple environmental stresses (23). First identified in cotton seeds during the late stages of embryogenesis (24), LEA proteins are widely expressed in plants. The sequences of LEA proteins are quite divergent but feature multiple copies of various low-complexity motifs that are used to classify them into different groups (25). Group-3 LEA proteins are of particular interest within the LEA protein family as they are found not only in plants (26–29) but also in diverse species of microbes and invertebrates (30–37). Consistent with being part of the group-3 LEA protein family, HeLEA1 homologs are found across diverse species, including bacteria, fungi, plants, and invertebrates, but not vertebrates (Fig. 1A and Table S2). Sequence alignment between HeLEA1 and representative homologs revealed not only reasonable sequence similarity but also long gaps (Fig. S2). The observation correlates with the high propensity for HeLEA1 homologs to be intrinsically disordered (83 of 96 with a mean IUPred disorder score > 0.5; Fig. 1B) and the tendency for LEA proteins to broadly feature multiple copies of sequence repeats that vary in number. We performed a motif search using the MEME algorithm (22) across 96 homologs and found conserved group-3 LEA protein motifs distributed along the HeLEA1 sequence with high-confidence (Fig. 1C and D). The motifs in HeLEA1 map reasonably well to the classic group-3 LEA motifs (38) (Fig. 1E). The amphipathic nature of the motifs is well conserved despite some variation in charge properties on the hydrophilic side (Fig. 1E).

Similar to its tardigrade homolog RvLEAM (14), HeLEA1 is also predicted to carry a mitochondrial targeting signal (MTS) at its N-terminus (Fig. S3). To characterize the secondary structure of HeLEA1, we expressed and purified a recombinant protein lacking the first 38 amino acids including the MTS (herein referred to as HeLEA1 unless otherwise noted) in *E. coli* and investigated its secondary structure using circular dichroism (CD). CD spectra of HeLEA1 showed characteristic signatures of random coil (RC), suggesting the protein is largely unstructured (Fig. 1F). We further characterized HeLEA1 by solution-state NMR spectroscopy using isotopically labeled proteins. Using a combination of standard triple resonance experiments and (^1^H-start) ^13^C-detect experiments, backbone resonances for 190 out of 200 residues (95%) in HeLEA1 were assigned at 278 K (Figs. 1G and S4). Two features stood out in the ^1^H_N_–^15^N 2D HSQC spectra: very narrow dispersion of amide proton chemical shifts; and clustering of the backbone ^1^H_N_,^15^N resonances in the 2D spectrum according to amino acid type. These features define HeLEA1 as an intrinsically disordered protein in solution (39) and are consistent with the lack of secondary structure shown in our CD spectra (Fig. 1F).

**Fig. 2.**
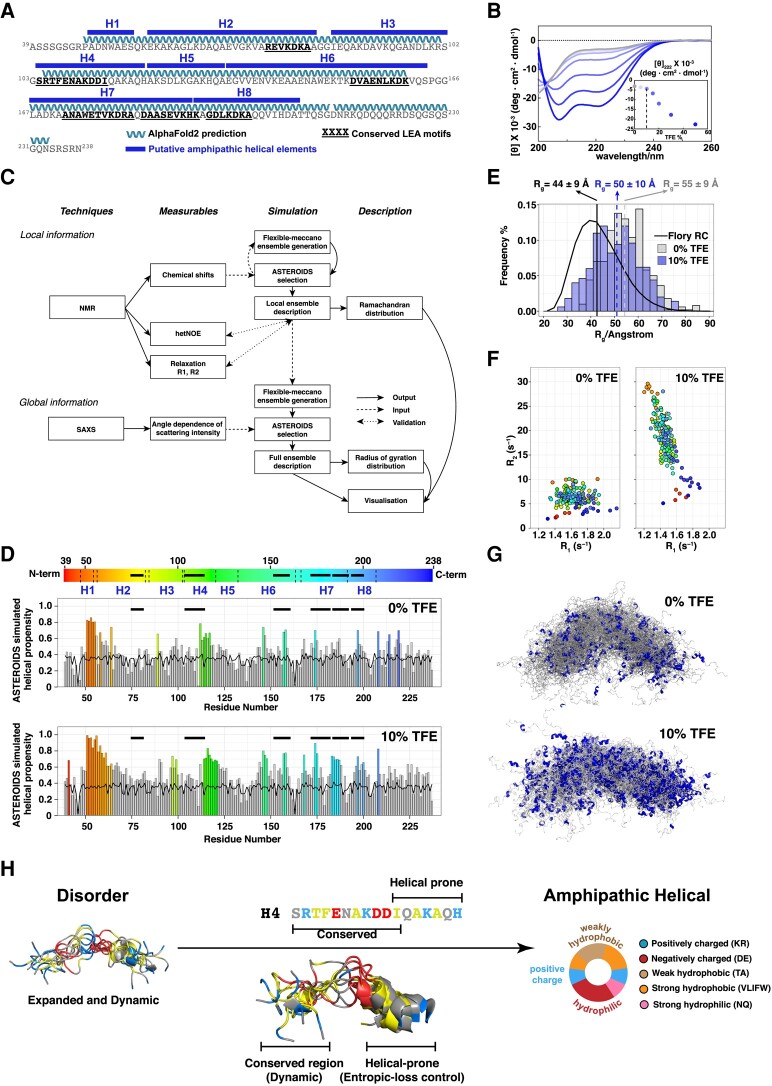
Dynamic disorder-to-helical transition in HeLEA1. A) Locations of putative amphipathic helical elements predicted by biophysical properties as described in Fig. S7 (upper bars), helical regions predicted by AlphaFold2 (ribbons), overlapping with conserved group-3 LEA motifs (bold and underlined) in HeLEA1. B) Titration of 10 μM HeLEA1 with increasing concentration of TFE monitored by CD. Inset shows the increase in helicity (indicated by molecular ellipticity at 222 nm) as a result of increased TFE concentrations. Note that at 10% TFE (dashed line), HeLEA1 appears predominantly disordered. C) Pipeline for integrative structural study of HeLEA1 in 0 or 10% TFE. NMR chemical shifts and SAXS data were used for describing local and global conformational features of HeLEA1. These experimental data were used as input for ASTEROIDS simulation to sample for the conformational ensemble that best fit the data. NMR relaxation data were further collected to provide additional insight into protein backbone dynamics and validate the ensemble description. D) Residue-specific helical propensity based on ASTEROIDS fits of NMR data of HeLEA1 in 0% TFE (top) or 10% TFE (bottom). Residues with significant helical propensity deviating >0.3 from RC (represented as the continuous horizontal line in the plot) are shown as colored bars. Bold horizontal bars represent conserved LEA motifs in Fig. 2A. E) Distribution of the radius of gyration (Rg) of the HeLEA1 ensemble generated assuming Flory RC behavior (solid curve, 44 +/– 9 Å) or fitted with SAXS data either from 0% TFE (gray bars, 55 +/– 9 Å) or 10% TFE (blue bars, 50 +/– 10 Å). F) Correlation maps of transverse (R_2_) and longitudinal (R_1_) relaxation rates of HeLEA1 in 0% (left) or 10% TFE (right). Ten per cent of TFE drastically increased the R_2_ relaxation rates in regions with increased helical propensity, colored with same color scheme as shown in Fig. 2D. G) Description of the conformational ensemble (100 representative conformers) of HeLEA1 in 0% TFE (left) and 10% TFE (right) by integrating NMR and SAXS data within the ASTEROIDS simulation pipeline. Helical regions in each conformer are highlighted in blue. H) Schematic depicting the disorder-to-helical transition for HeLEA1 H4 as an exemplar: weak amphipathic elements comprise a region of higher disorder propensity adjacent to a region with an increase in intrinsic helical propensity. This arrangement may reduce the entropic cost of the disorder-to-helical transition. Ten conformers for the H4 region as predicted from ASTEROIDS ensembles are shown.

In addition to the predicted disorder in solution (Fig. 1B) and group-3 LEA motifs (Fig. 1E), our sequence search also suggested that some HeLEA1 homologs harbor sequence motifs with similarity to the apolipoprotein superfamily (Pfam) (Fig. S5). Moreover, a PhyRE2 (40) structural similarity search probing the PDB database with the HeLEA1 sequence yielded hits with apolipophorin-III, α-synuclein, and HSP12 (Fig. S6), all of which associate with membranes via amphipathic helices (41). While our CD and NMR data suggest HeLEA1 lacks stable helical conformation in solution, we speculated that HeLEA1 may have a weak helical propensity and can undergo disorder-to-helical transition like other LEA proteins in addition to the high-confidence LEA motifs (28). We, therefore, devised a computational pipeline to identify potential 3–11 helical elements (minimum length = 7 residues, with aligned amphipathic interface) that may interact with lipids according to previously reported criteria for predicting continuous amphipathic helical elements (42) and apolipoprotein motifs (43, 44) (Fig. S7). We identified eight putative amphipathic 3–11 helical elements of various lengths in HeLEA1 (H1–H8, Figs. 2A and S8). These elements are enriched in weakly hydrophobic residues (alanine and threonine) and exhibit a small hydrophobic surface (between 3 and 5 of the 11 projected positions are hydrophobic). Moreover, four of the eight helices (H2, H6–H8) displayed strong enrichment of positively charged residues (lysine and arginine) at the boundary of the hydrophobic and hydrophilic surfaces and enrichment of negatively charged residues (glutamates and aspartates) in the hydrophilic surface. The remaining helices (H1, H3–H5) also exhibit these features, albeit more weakly. The biophysical prediction corresponds well with PSIPRED (Fig. S9) and AlphaFold2 prediction (45) (Figs. 2A and S8). The low pLDDT score of the AlphaFold2 prediction suggests a low confidence global structure (Fig. S8), probably due to the disordered nature of HeLEA1 causing difficulty with sequence alignment (Figs. 1B and S2). Nonetheless, local secondary structural elements predicted by AlphaFold2 match our biophysical prediction (Fig. S8). The low confidence in stably folded long amphipathic helices but agreement on local secondary structure predictions would be consistent with a dynamic disorder-to-helical transition in HeLEA1, a common feature of IDPs binding to membranes (46–49) that is also shared by other group-3 LEA proteins (23).

### Group-3 LEA protein motifs in HeLEA1 remain conformationally dynamic during the early stages of its disorder-to-helical transition

Our computational analysis of HeLEA1 suggests that specific sequence elements can adopt both disordered and structurally defined states largely consisting of amphipathic elements (Figs. 2A and S8). To gain structural insight into these elements, we sought to probe HeLEA1's conformational states and backbone dynamics in solution, using trifluoroethanol (TFE) perturbation. TFE is frequently used in protein folding studies to mimic conditions under which transient helical components of the conformational ensemble are stabilized (50). CD revealed that TFE indeed induced helicity of HeLEA1 in a concentration-dependent manner (Fig. 2B). We note that on an ensemble level, at low TFE concentration (10%), HeLEA1 remained predominantly disordered and did not yet get through the cooperative folding stage of the S-shaped transition (Fig. 2B, inset). We also note that at a low concentration of TFE (10%), there is likely to be minimal impact on local helix–helix interactions and long-range interactions that may involve water as a solvent (50). This is important in that at 10% TFE, the protein ensemble is not artificially forced to shift to a fully folded state but rather represents an early stage of the transition within a relatively mild environment that may stabilize helicity. Therefore, conformational differences observed under 10% TFE compared to 0% TFE likely provide insight into disorder-to-helical transitions of the amphipathic elements in HeLEA1. Accordingly, we performed integrative structural analyses at 0 and 10% TFE conditions (Fig. 2C–G), using a combination of small angle X-ray scattering (SAXS) and NMR to provide global and residue-specific structural information, respectively. We employed the *ASTEROIDS* algorithm (51, 52) to determine the conformational ensemble that best described all experimental data (Fig. 2C) simultaneously. In agreement with the predicted helicity (Fig. 2A), various regions in the HeLEA1 sequence show a local helical propensity in their secondary chemical shifts (Fig. S10). While 10% TFE mildly increases global helical propensity, certain regions of H1 (51–54), H2 (55–60), H4 (114–120), and H7 (183–187) exhibit a significant increase in helical conformation (Figs. 2D and S10). The radii of gyration (*R_g_*) of HeLEA1 in 0% TFE and 10% TFE were larger than the *R_g_* predicted for a statistical RC (Fig. 2E), and the addition of 10% TFE only resulted in a small decrease of *R_g_* (Figs. 2E and S11, Table S3). These results correspond well with HeLEA1 occupying a largely expanded disordered state but with a significant increase in helical propensity within short local segments at the early stage of disorder-to-helical transition (Fig. 2G).

To better understand this dynamic transition, we investigated the protein backbone dynamics as HeLEA1 undergoes conformational change through a disorder-to-helical transition by ^15^N-NMR relaxation measurements. ^15^N{^1^H} heteronuclear nuclear Overhauser effect (hetNOE) experiments that probe fast (picoseconds) backbone dynamics revealed a small global increase in 10% TFE, indicating a modest increase in overall rigidity (Fig. S12). Similarly, a modest decrease was observed for ^15^N *R*_1_ (1/*T*_1_) longitudinal relaxation rates (Fig. 2F). In contrast, ^15^N *R*_2_ (1/*T*_2_) transverse relaxation rates displayed a dramatic increase under 10% TFE perturbation (Fig. 2F). While the general increase of *R*_2_ across HeLEA1 can be a result of increased solvent viscosity (53), sequence elements within regions of increased helicity displayed a large and specific increase in *R*_2_ (Fig. 2D and F). This observation corresponds well with previous reports where the *R*_2_ relaxation rates for residues with “segmental motions” associated with the formation of transiently populated secondary structural elements in an IDP are exquisitely sensitive to changes in viscosity (54). We hypothesized that a disorder-to-helical transition is initiated via the population of local secondary structure elements as a response to an increase in solvent viscosity, resulting in a global decrease in backbone flexibility and shifts of the overall conformational ensemble toward higher helical propensity. Importantly, the regions that mapped to evolutionarily conserved LEA motifs did not completely overlap with regions of higher helical propensity (Fig. 2D). The localizations of these conserved conformationally dynamic motifs imply that maintaining a dynamic disordered state in the amphipathic helical elements may be important in the function of LEA proteins, while the neighboring helical-prone regions may buffer the entropic cost of the disorder-to-helical transition (Fig. 2H).

### HeLEA1 binds to negatively charged membranes through dynamic disorder-to-helical transition

**Fig. 3.**
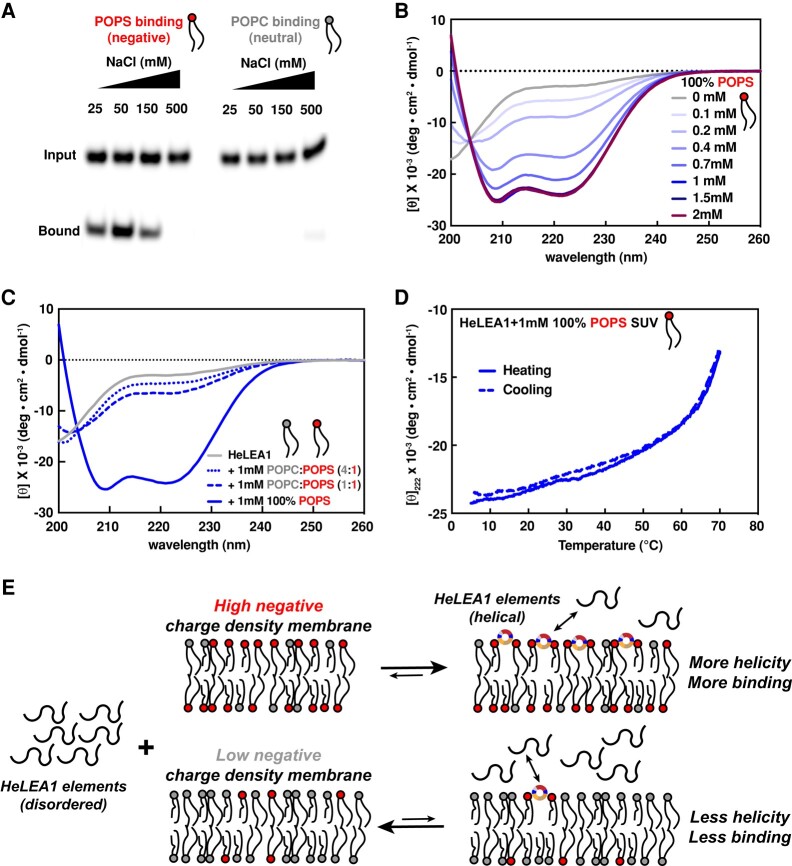
HeLEA1 binds to negatively charged membrane through dynamic disorder-to-helical transition. A) Lipid flotation assay with Alexa Fluor 488–labeled HeLEA1 and liposomes made of various lipids (POPS, left; POPC, right) at different salt concentrations. B) Titration of 10 μM HeLEA1 with increasing amounts of 100% POPS SUVs monitored by CD. C) Titration of 10 μM HeLEA1 with 1 mM SUVs of different ratios of negatively charged POPS versus neutral POPC, monitored by CD. Decreasing the amount of POPS fraction significantly reduced the induced helicity of HeLEA1. D) Thermal melting and refolding curves monitoring dynamics of helicity of 10 μM HeLEA1 induced by 1 mM POPS SUVs. The disorder-to-helical transition is completely reversible up to 70 °C. E) Schematic depicting the dynamic disorder-to-helical transition of HeLEA1 induced by negatively charged membrane. Sequence elements in HeLEA1 is intrinsically disordered in solution and go through disorder-to-helical transition into amphipathic helical conformation induced by binding to negatively charged head groups of lipids in membranes. The conformational change and binding are dynamic and reversible. The amount of binding and induced helicity is correlated to the negative charge density on the membrane.

As group-3 LEA proteins are generally proposed to stabilize biological membranes (23), we directly tested whether HeLEA1 can be recruited to membranes using a liposome flotation assay. As the predicted amphipathic helical elements in HeLEA1 are enriched in positively charged residues (Figs. S8 and 2A), we tested HeLEA1 recruitment to small unilamellar vesicles (SUVs) made with either negatively charged POPS lipid or neutral POPC lipid. At physiological salt concentrations, HeLEA1 was stably associated with POPS, but not POPC SUVs (Fig. 3A). Increasing the salt concentration, which suppresses electrostatic interactions, abolished the binding of HeLEA1 to negatively charged liposomes and had a minor effect on the interaction with neutral liposomes (Fig. 3A). We, therefore, reasoned HeLEA1 can bind negatively charged membranes primarily through electrostatic interactions.

We next determined whether membrane binding induces any structural changes. We titrated HeLEA1 with SUVs containing various ratios of neutral lipids to negatively charged lipids and monitored the changes in protein secondary structure by CD. HeLEA1 exhibits increased helicity with increasing concentrations of negatively charged SUVs (Figs. 3B and S13A and B), and the increased helicity is proportional to the fraction of negatively charged lipids (Fig. 3C). CD spectra of the POPS titration revealed an isodichroic point at 204 nm, which is characteristic of a classic disorder-to-helical transition (61), and similar to that observed for TFE titration (Fig. 2B). Deconvolution of the CD titration curves with Bestsel (62) suggested that at least 60% of the residues in lipid-bound HeLEA1 are in a helical conformation, corresponding well with predictions of possible secondary structures (Fig. 2A). The CD spectra at low SUV concentrations resemble the CD spectrum of the 10% TFE ensemble during the initial stages of the disorder-to-helical transition (Fig. S13C). However, due to contributions from both electrostatic and hydrophobic interactions, SUVs exhibit a significantly stronger capability to stabilize the helical conformation of HeLEA1 at higher SUV concentrations (Fig. 3B). Moreover, we observed complete reversibility without hysteresis of such membrane-induced disorder-to-helical transition in thermal melting and refolding experiments up to 70 °C (Fig. 3D). While both thermal melting of secondary structures in HeLEA1 and changes to SUV properties (e.g. increased membrane fluidity) may contribute to the loss of the helicity as temperature increases, the results confirm that the disorder-to-helical transition of HeLEA1 induced by negatively charged membrane binding reacts in a fast and dynamic manner to changes in the environment without metastable intermediate state. This corresponds well with a uniform distribution of weak amphipathic helical elements throughout HeLEA1 (Fig. 2A) and the NMR dynamics data (Fig. 2F). Importantly, the positional distribution of the conserved dynamic LEA motifs correlates with a weak local hydrophobic moment and the presence of negative charge clusters featuring a low lipid discrimination factor, implying weak protein–membrane interaction (Fig. S14), which is also consistent with deviations in charge properties of residues on the hydrophilic interface of LEA motifs from classic group-3 LEA motifs (Fig. 1E). The sequence features highlight the importance of weak and dynamic membrane binding in the function of HeLEA1 and the preference of negatively charged membrane in bilayer-induced disorder-to-helical transition (Fig. 3E).

### HeLEA1 stabilizes negatively charged membranes

**Fig. 4.**
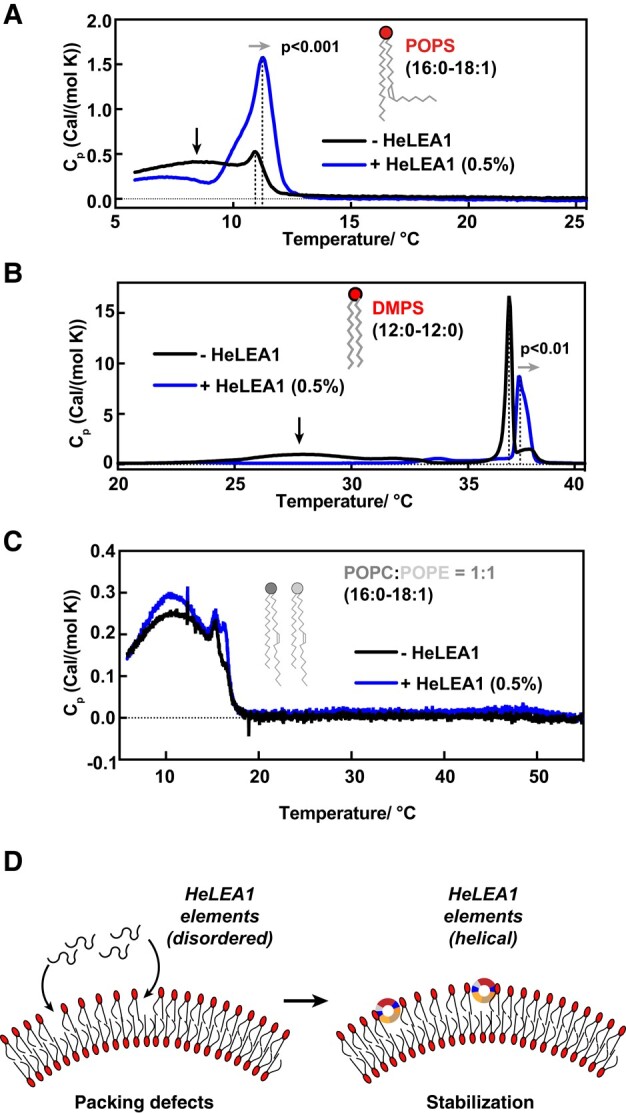
HeLEA1 increases phase transition temperature of negatively charged membranes. A–C) DSC for A) POPS, B) DMPS, C) POPC-POPE SUVs without (black) and with HeLEA1 (0.5% molar ratio, blue). HeLEA1 suppressed thermally induced phase transition at low temperature (broad peaks indicated by dark arrows) and increased phase transition temperature (peak position; dashed lines, and light arrows) for negatively charged membranes (POPS and DMPS) regardless of side chain saturation but has minor effect on neutral membranes (POPC and POPE). The statistical significance in Fig. 4A and B was determined by an unpaired t test (*n* = 3). D) Proposed mechanism for the stabilization effect of HeLEA1. A disorder-to-helical transition occurs within putative amphipathic elements in HeLEA1 when HeLEA1 interacts with negatively charged lipids at areas of the membrane with lipid packing defects, resulting in stabilization.

Based on these data, we postulated that weak binding of HeLEA1 to negatively charged membranes can modulate the biophysical properties of lipid bilayers. We first tested this model by investigating the temperature-dependent lipid phase transition of synthetic membranes using differential scanning calorimetry (DSC) in the presence or absence of HeLEA1. DSC measures heat flux toward the sample as a result of changing temperature. A lipid bilayer will have a characteristic DSC peak triggered by changes to the packing of fatty acids side chains with increasing temperature. In the absence of HeLEA1, negatively charged POPS SUVs displayed a complex phase transition profile over a broad temperature range (Fig. 4A), possibly due to packing defects induced by unsaturation and high curvature of SUVs as previously reported (55–57). The addition of substoichiometric amounts of HeLEA1 (1:200 molar ratio of protein:POPS) suppressed phase transition at low temperatures, promoted cooperativity in the phase transition as reflected by the sharper peak (Fig. 4A), and resulted in a small but significant increase of the dominant phase transition temperature (10.96 ± 0.04 to 11.28 ± 0.03 °C, *n* = 3, *P* = 0.0005; dashed lines in Fig. 4A). This corresponds well with previously reported observations where membrane stress and packing defects in SUVs have been shown to be stabilized by IDPs like alpha-synuclein (58). Experiments using SUVs composed of 100% DMPS, a lipid with saturated and shorter fatty acid chains that tend to induce fewer packing defects than POPS, similarly increased the dominant phase transition temperature (36.78 ± 0.03 to 37.24 ± 0.08 °C, *n* = 3, *P* = 0.001), but had less effect on cooperativity of the transition (Fig. 4B). In contrast, the phase transition behavior of SUVs composed of neutral lipids POPC and POPE remained mostly unchanged in the presence of HeLEA1 (Fig. 4C), despite this mixture tending to induce more packing defects (56, 57). The DSC results suggest that HeLEA1 can stabilize lipid bilayers against curvature stress and packing defects (Fig. 4D). The charge dependence of the stabilization corresponds well with the preference of HeLEA1 to bind negatively charged, coupled with its disorder-to-helical transition (Fig. 3E).

**Fig. 5.**
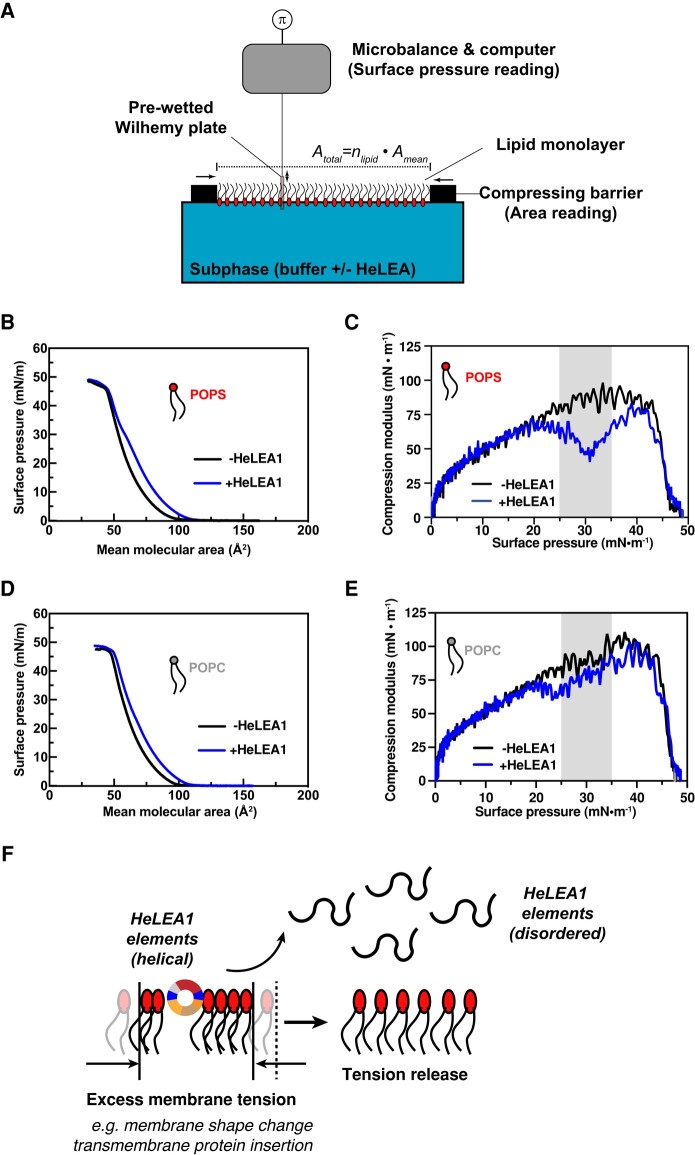
HeLEA1 buffers excess surface tension in lipid monolayer. A) Schematics illustrating the instrument setup for measuring lipid monolayer compression isotherms. Mean molecular area *A*_mean_ and surface pressure *π* are plotted in B and D. The subsequently calculated $C_{S}^{\;\;-1}$ values are reported in C and E. B) Compression isotherm of POPS monolayer with or without 3 nM HeLEA1 in subphase (1:50 protein:lipid ratio). C) Change of $C_{S}^{\;\;-1}$ values in POPS monolayers with respect to surface pressure. Shaded areas depict the range of physiological surface pressure. D) Compression isotherm of POPC monolayer with or without 3 nM HeLEA1 in subphase (1:50 protein:lipid ratio). E) Change of $C_{S}^{\;\;-1}$ values in POPC monolayers with respect to surface pressure. Shaded areas depict the range of physiological surface pressure. F) Proposed mechanism for tension release by HeLEA1. When excess surface tension occurs at a HeLEA1-bound membrane, HeLEA1 elements would unfold and detach from the membrane to buffer such tension, stabilizing the membrane and increasing membrane fluidity.

We next tested if HeLEA1 can also modulate mechanical properties of membranes using Langmuir-monolayer methodology, in which the relationship between the surface pressure and area of a lipid monolayer is determined as compression isotherms (Fig. 5A). The resulting curves are used to compute the compression modulus $\left(C_{S}^{\;\;-1}\right)$, which reports on the stiffness of the lipid monolayer: lower $C_{S}^{\;\;-1}$ values correspond to softer and more compressible membranes with increased fluidity (60). Despite its negligible surface property (Fig. S15), the presence of 3 nM HeLEA1, which translates to a protein-to-lipid ratio of 1:50, shifted the compression isotherm of POPS monolayer to the right, corresponding to HeLEA1 localizing to the air–water interface (Fig. 5B). The presence of HeLEA1 substantially decreased $C_{S}^{\;\;-1}$ (i.e. increased membrane fluidity) for POPS monolayer (Fig. 5C), demonstrating that HeLEA1 directly interacts with the POPS monolayer and changes its mechanical property. Notably, this effect was most prominent in the physiological range of surface pressures, i.e. 25–35 mN/m (Fig. 5C, gray box) (60). The mean $C_{S}^{\;\;-1}$ of the POPS monolayer changed from 85.4 ± 5.8 to 54.8 ± 6.6 mN/m at physiological membrane surface pressure, suggesting a significant increase in membrane fluidity and compressibility (60). At high surface pressure, the $C_{S}^{\;\;-1}$ of the POPS monolayer with HeLEA1 was restored to that without HeLEA1, suggesting that HeLEA1 lost contact with the lipid monolayer as a result of passive exclusion of HeLEA1 molecules at air–water interface due to high surface pressure. In contrast, while the $C_{S}^{\;\;-1}$ of POPC monolayer titrated with HeLEA1 also exhibits similar changes at sufficiently high surface pressure, it only exhibits marginal change within physiological surface pressure (Fig. 5E). These observations suggest that modulation of membrane compressibility by HeLEA1 under physiological surface pressure is dependent on its preferential recruitment to negatively charged phospholipids, but not neutral lipids, through disorder-to-helical transition (Fig. 3E). Our observations demonstrated that when negatively charged lipids within a bilayer are loosely packed at the air–water interface, HeLEA1 tends to associate with the lipids and adopts a helical conformation, while excess surface tension occurring at a HeLEA1-bound membrane triggers unfolding of HeLEA1, facilitating protein detachment from the membrane, which results in a buffering effect of excess tension (Fig. 5F).

### HeLEA1 expression enhances tolerance to hyperosmotic stress on nonfermentable carbon sources

**Fig. 6.**
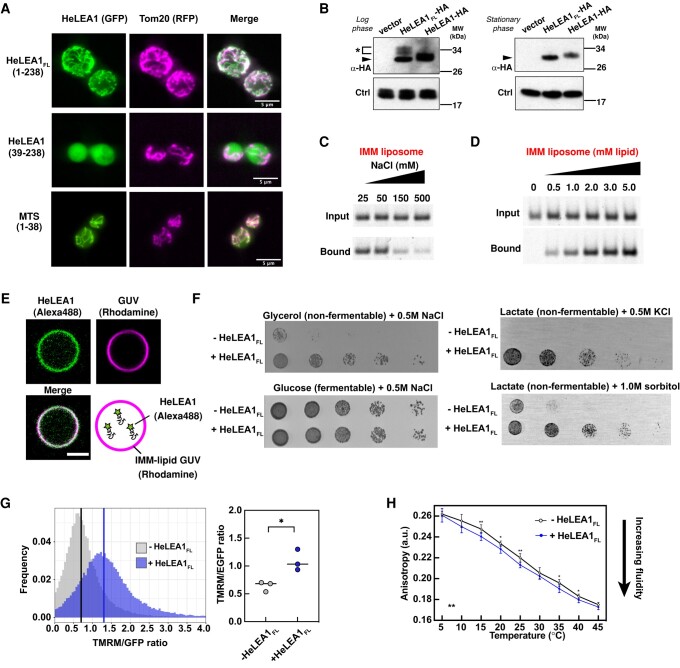
Physiological impact of HeLEA1 expression. A) GFP-tagged (C-terminal) full-length HeLEA1 (HeLEA1_FL_-GFP) colocalized with mitochondria (marked by Tom20-RFP) when expressed in yeast; removal of the N-terminal 38 amino acids corresponding to the predicted MTS (HeLEA1-GFP) resulted in cytoplasmic localization; fusing the predicted MTS to GFP also resulted in mitochondrial localization (MTS-GFP). B) Immunoblotting of log-phase (left) and stationary-phase (right) cells expressing HA-tagged HeLEA1_FL_ or HA-tagged HeLEA1. The blots revealed three species for log-phase cells expressing HeLEA1_FL_ that likely correspond to full-length HeLEA1, MPP-cleaved HeLEA1 (bands marked by *), and mature HeLEA1 (triangle) with additional processing by mitochondrial processing enzymes; and one single mature HeLEA1 band for stationary-phase cells. HeLEA1-HA migrated as a single band of intermediate molecular weight in both cases. In either case, Erv46p was used as a loading control. C) Lipid flotation assay with Alexa Fluor 488–labeled HeLEA1 and liposomes made of a composition mimicking that of the IMM at different salt concentrations. The concentration of liposomes was kept the same at 1 mM. D) Lipid flotation assay with Alexa Fluor 488–labeled HeLEA1 and IMM liposomes at different concentrations. Salt concentration was kept the same at 50 mM. E) Confocal imaging shows colocalization of HeLEA1 and GUVs with a composition mimicking the IMM. GUVs were labeled with DOPE-lissamine rhodamine. HeLEA1 was labeled with Alexa Fluor 488 and encapsulated inside GUVs. Scale bar: 3 μm. F) HeLEA1_FL_ expression had a positive fitness effect on various hyperosmotic stress when cells were grown on nonfermentable carbon sources (glycerol and lactate), in which mitochondrial activity is crucial for cell survival, but not with fermentable carbon source (glucose) where mitochondrial activity is less critical for survival. G) Normalized TMRM fluorescence for yeast cells with or without HeLEA1_FL_ expression grown on glycerol. HeLEA1_FL_ expression significantly increases mitochondrial membrane potential, suggesting enhanced mitochondrial activity in live cells. Representative cell sorting data for one replica (left, lines indicate the median of the distribution) and statistical test for three independent replicas (*P < 0.05, Welch t test, *n* = 3, right; each replica has at least 60,000 cells) are shown. H) Temperature-dependent fluorescence anisotropy of DPH-stained mitochondria from yeast cells grown in normal media. Mitochondria from yeast expressing HeLEA1_FL_ had lower anisotropy than mitochondria from yeast not expressing HeLEA1_FL_ (**P < 0.01, two-way ANOVA, *n* = 7, error bars represent the SD), suggesting that HeLEA1_FL_ expression increases the membrane fluidity of mitochondria. For each temperature pair, an independent unpaired t test was also performed, and the statistical significance was indicated (*P < 0.05, **P < 0.01, two-tailed, unpaired t test, *n* = 7).

To further explore the physiological impact of HeLEA1, we first followed the subcellular localization of full-length HeLEA1, including the predicted MTS (Fig. S3), now termed HeLEA1_FL_, by fusing GFP to its C-terminus and expressing the fusion construct in *S. cerevisiae*, which lacks HeLEA1 orthologs. HeLEA1_FL_ colocalized with the mitochondrial marker Tom20 (63), whereas HeLEA1 lacked the putative MTS accumulated in the cytoplasm. Appending the predicted MTS to GFP resulted in mitochondrial localization, suggesting the predicted MTS is both necessary and sufficient for mitochondrial targeting (Fig. 6A). Consistent with delivery to the mitochondrial matrix, immunoblot analysis of yeast lysates from midlog phase cells expressing HA-tagged HeLEA1_FL_ or HeLEA1 revealed multiple bands for HeLEA1_FL_, the smaller of which comigrated with the single HeLEA1 band (Fig. 6B). Immunoblot of yeast lysates from stationary-phase cells revealed a single band HeLEA1_FL_ corresponding to a slightly smaller molecular weight compared to HeLEA1 (Fig. 6B). These observations are consistent with HeLEA1_FL_ being targeted to the mitochondrial matrix, then cleaved by the mitochondrial processing peptidase (MPP), with possible further processing by other mitochondrial processing enzymes (64).

Inspired by the mitochondrial matrix localization, we next tested if HeLEA1 can be recruited to membranes with more physiologically relevant lipid composition. We generated SUVs that mimicked the inner mitochondrial membrane (IMM) composition (65) and tested HeLEA1's recruitment using a liposome floatation assay. We observed that similar to negatively charged POPS vesicles, HeLEA1's interaction with IMM liposome also showed an anticorrelation with increasing salt concentration (Fig. 6C), possibly due to IMM composition consisting of a significant fraction of negatively charged lipids including phosphatidylinositol and cardiolipins. We also observed a stable increasing amount of HeLEA1 recruitment and saturation at higher concentrations of IMM liposomes, suggesting a quantitative binding event (Fig. 6D). We further encapsulated N-terminally fluorescently labeled HeLEA1 inside giant unilamellar vesicles (GUVs) made of IMM composition and imaged both lipid and protein by confocal imaging. HeLEA1 colocalized with the GUV membrane (Fig. 6E), corresponding well with the results from liposome floatation assays.

It has been reported that expression of HeLEA1 was upregulated when its host species *H. exemplaris* was subject to desiccation stress (9) that may induce various membrane stresses (66). Moreover, characterizations of another tardigrade homolog of HeLEA1, RvLEAM, revealed that it localizes to mitochondria and may enhance hyperosmotic stress tolerance in mammalian cells (14). We speculated that HeLEA1 may confer a similar phenotype in yeast through interaction with IMM. As the metabolic requirement for mitochondrial function is only necessary for yeast growth under nonfermentable conditions (67), we characterized yeast growth phenotypes under a combination of osmotic stress with different carbon sources. We found that HeLEA1_FL_ expression conferred a substantial growth advantage when grown on nonfermentable glycerol media at 37 °C, where cell growth is dependent on mitochondrial function (Fig. 6F). In contrast, on glucose media, where mitochondrial biogenesis is repressed and mitochondrial function is not essential, there was no growth advantage (Fig. 6F). We observed similar effects in cells grown on lactate, another nonfermentable carbon source, and using other hyperosmotic stress conditions including 0.5 M KCl and 1.0 M sorbitol (Figs. 6F and S16). Additionally, when we stained midlog phase cells growing in nonfermentable glycerol with TMRM, a fluorescent dye whose accumulation in mitochondria is dependent on membrane potential, we found that HeLEA1_FL_ expression resulted in higher TMRM fluorescence (normalized to mitochondrial marker TOM20-eGFP), consistent with increased mitochondrial membrane potential (Fig. 6G). Recently, it has been reported that there is a dynamic relationship between the fluidity of IMM and cellular respiration (68). Inspired by this report and the biophysical effects of HeLEA1 on lipid bilayers (Figs. 4D and 5F), we sought to test if HeLEA1 can also affect the membrane properties of mitochondria. We purified mitochondria from yeast cells with or without expression of HeLEA1_FL_ and stained them with diphenylhexatriene (DPH). DPH fluoresces in a hydrophobic environment, and its anisotropy reports on membrane fluidity, where low anisotropy (faster tumbling) corresponds to a more fluid membrane, creating a useful reporter for global membrane properties. Mitochondria from HeLEA1_FL_-expressing cells showed small but significant changes in temperature-dependent DPH anisotropy, i.e. altered membrane properties, compared to wild-type cells (Fig. 6H). These phenotypes correspond well with the mitochondrial localization of HeLEA1_FL_ (Fig. 6A) and HeLEA1's biophysical role in the stabilization of biological membranes (Figs. 4D and 5F).

### Sequence features of group-3 LEA proteins correlate with their localizations

**Fig. 7.**
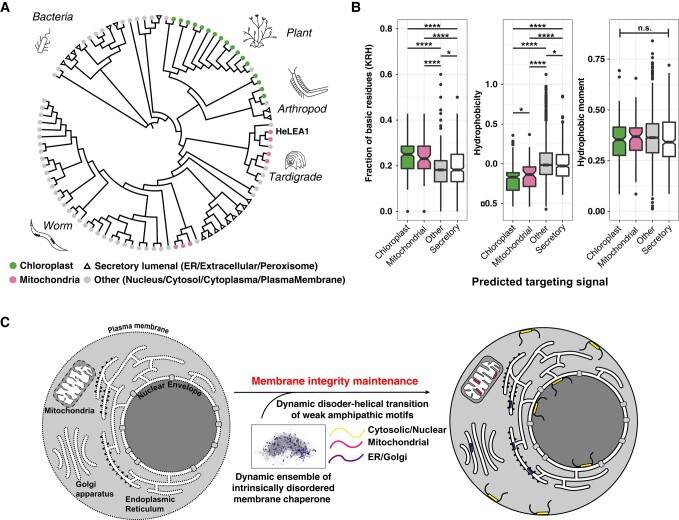
Evolution of group-3 LEA proteins correlates with predicted localizations. A) Evolutionary tree of HeLEA1 homologs annotated by their predicted subcellular localization. Several HeLEA1 homologs localize to various membrane-bound organelles. HeLEA1 was predicted to localize to the mitochondria. B) Amphipathic elements in HeLEA1 homologs carrying different predicted targeting signals share different compositions in basic residues and hydrophobicity but with the same hydrophobic moment. (*P < 0.05, ****P < 0.0001; n.s., not significant across all entries, Kruskal–Wallis rank sum test). The amphipathic elements are identified using the algorithm described in Fig. S7. Each box represents the interquartile range (IQR) of the dataset, whiskers represent plus/minus 1.5 IQR from the box hinge, and outliers are plotted as dots. Sample size: chloroplast: 220, mitochondrial: 75, secretory: 447, other: 2515. C) Proposed function for HeLEA1 homologs. HeLEA1 homologs adapt their sequences to their designated subcellular localizations and may confer various stress-tolerance phenotypes by acting as modulators for membrane biophysical properties.

The localization of HeLEA1 to mitochondria prompted us to perform a comprehensive computational prediction of localizations of its homologs. Our predictions suggested that different HeLEA1 homologs carry specific targeting signals localizing to distinct subcellular compartments and organelles, including the chloroplast (for plants), mitochondria, secretory pathway, and nucleus (Fig. 7A and Table S4). The diverse localizations of the homologs to various membrane-bound organelles correspond well with proposed functions involving bilayer modulation. As the lipid composition of different organelles varies in the cell (57, 69), we wondered if the sequence features of the helical elements in HeLEA1 homologs change with respect to their localizations. Indeed, we found that helical elements in mitochondrial and chloroplast homologs tend to have a higher fraction of basic residues and less hydrophobic residues, compared to homologs carrying signal peptides or having cytoplasmic/nucleic localizations (Fig. 7B). This change in amino acid distribution correlates well with the lipid compositions of the respective organelles, in that mitochondria and chloroplasts contain more negatively charged lipids including cardiolipin (enriched in IMM) and sulfoquinovosyl diacylglycerol lipids (enriched in thykaloid membrane), while lacking cholesterol (70). Importantly, despite the variance in amino acid composition, the hydrophobic moments for the helical elements in different homologs do not show a significant difference (Fig. 7B). These results suggest that the sequence of HeLEA1 homologs diverges to adapt to different localizations but conserves their biophysical activity.

## Discussion

Since the discovery of LEA proteins from cotton seeds, the relevance of these IDPs for diverse abiotic stress tolerance has been demonstrated in various organisms and model systems from different kingdoms (systematically reviewed in Ref. (23)), suggesting conserved mechanisms of action despite sequence divergence. In vitro studies of LEA proteins from different families demonstrate a wide spectrum of molecular action, including protecting proteins from denaturation and aggregation (33, 71, 72), scavenging metal ions (73), stabilizing sugar glasses in anhydrobiotic tissues (74, 75), and maintaining integrity and structure of biological membranes (76–78). Among them, group-3 LEA proteins are particularly interesting due to their presence in diverse organisms including animals (30). Sequence analysis and biophysical studies on various members from this family, including COR15A/B from *Arabidopsis* (27–29), CeLEA1 from *Caenorhabditis elegans* (32), AavLEA1 from *Aphelenchus avenae* (33), AfrLEA2, and AfrLEA3m from *Artemia franciscana* (34, 35), and PvLEA1-3 from *Polypedilum vanderplanki* (36, 37), suggest that such protection may be meditated through maintenance of the integrity of biological membranes, associated with a possible amphipathic helical conformation of LEA motifs in these proteins. Despite extensive functional and biophysical studies on a subset of group-3 LEA proteins (30–37), how the evolutionary conservation and divergence in the sequences of this protein family are translated into their conformational features and functions still remains to be clearly elucidated. Herein, using the HeLEA1 protein from tardigrade as a model, we performed a comprehensive structural, biophysical, and evolutionary analysis to determine the key features that contribute to its molecular function.

We showed that HeLEA1 is an IDP that undergoes disorder-to-helical transition in the presence of negatively charged lipids. Such membrane-induced disorder-to-helical transitions have been extensively studied for sequence motifs from different LEA protein families and have been demonstrated to play an important role in membrane stabilization (27–37, 76–78). However, the evolutionary pressure to maintain a primarily disordered state remained to be fully elucidated. IDPs are frequently proposed to function as an entropic buffer, maintaining a degree of disorder even when adopting a functionally relevant conformational state (8). The high conformational entropy of the intrinsically disordered state stabilizes HeLEA1 in solution, whereas HeLEA1 folding upon binding to negatively charged lipids balances a loss of conformational entropy for enthalpic gain from electrostatic interactions, exposing the hydrophobic surfaces of the weakly amphipathic helical elements. Such dynamic interactions may buffer both loose lipid packing, for example packing defects associated with membrane curvature (Fig. 4D), and crowded lipid packing, which may stem from excess surface tension (Fig. 5F). The observed effects of HeLEA1 on synthetic membranes imply that HeLEA1 as an IDP does not monotonically promote or prevent lipid packing, but rather tends to maintain the physiological properties of biological membranes under different types of membrane stress, thereby facilitating homeostasis of lipid packing. The small increase of mitochondrial membrane fluidity as a result of HeLEA1 expression likely reflects an ensemble average (over complex and heterogeneous membrane environments) of the effect that HeLEA1 has on the mitochondrial membrane. These helical elements in HeLEA1 and its close homologs differ from amphipathic elements observed in dehydrins which are strongly positively charged and bear larger hydrophobic moments (78–80) that generally correlate with stronger membrane interaction and deeper insertion (57). The differences in biophysical properties of these elements may also explain why HeLEA1 increases the phase transition temperature of bilayers, whereas dehydrins are reported to decrease the phase transition temperature of dried or frozen lipids (78–80). Importantly, the evolutionarily conserved LEA motifs tend to possess a high-disorder propensity during the conformational change (Fig. 2H), highlighting the importance of protein intrinsic disorder being selected even when the functional state is partially folded.

Our evolutionary analyses show that while amphipathic elements in HeLEA1 homologs display trade-offs between the fraction of basic residues and hydrophobic residues to adapt to the lipid composition of their diverse subcellular location (57, 69) (Fig. 7A and B), the hydrophobic moments that correlate to the interaction strengths with lipid bilayers remain unchanged (Fig. 7B). We thus propose that the molecular function of weak lipid binding (i.e. low hydrophobic moments) is conserved, but the mechanism for lipid recognition (electrostatic-driven vs. hydrophobic-driven) may be fine-tuned according to the relevant subcellular locations, where different membrane-bound organelles differ in their lipid compositions (57, 69), as well as pH in local environment (81, 82). Previous work on Lti30 from the dehydrin family of LEA proteins has demonstrated histidine residues in sequence motifs playing an important role in pH-regulated membrane binding (80). Proteins with distant structural similarity to HeLEA1, such as apolipoproteins, may have evolved away from the need to protect organelles from environmental stresses and acquired new functions, such as stabilization of lipoprotein particles in mammals. When compared to similar amphipathic elements observed in apolipoproteins that are known to stably bind to lipids for structural scaffolding, HeLEA1 homologs display a weak hydrophobicity and local hydrophobic moment, higher disorder score, and a low lipid discrimination factor (Fig. S17). These features correspond well with an evolutionary trajectory to change their biophysical properties from dynamic and disordered to stable and folded.

Heterologous expression of IDPs from abiotic stress-tolerant organisms has been linked to multiple molecular mechanisms. Extensive studies on IDPs from extreme-stress-tolerant organisms tardigrade suggest multiple molecular mechanisms may be used to combat extreme stresses, including CAHS proteins adopting a vitrified state or formation of fibrous gels upon stress that might chaperone cellular proteins (9, 10) and modulating vitrification of small molecules like sugars (11, 12). The presence of group-3 LEA proteins in multiple tardigrade species may represent an alternative molecular strategy for extreme abiotic stress tolerance (14, 15). We found that HeLEA1 carries a targeting signal that confers proper mitochondrial localization for mitochondrial-related stress-tolerance phenotypes (Fig. 6), suggesting that subcellular localization might also be important in specification of IDP function, working in concert with sequence biophysical properties (Fig. 7B). While organelle-specific functional mechanisms for these LEA proteins remain to be further elucidated, such a general mechanism for modulating membrane biophysical properties corresponds well with previously suggested functions for group-3 LEA proteins (23). The targeting signal in HeLEA1 homologs ensures proper delivery of such proteins into subcellular membranes that are not in direct contact with the cytosol, such as IMM, chloroplast inner membrane and thykaloid membrane, and inner nuclear membrane. Moreover, the lipid composition and the optimal biophysical properties of each subcellular organelle may also change during distinct stresses (83), which may require different flavors of amphipathic helical elements in various LEA protein families and may explain the differences in functions and sequences between HeLEA1 and dehydrins (78–80). The adaptation to the variations in the environment may explain why the copy numbers and exact LEA motif sequences vary among the motifs from homologs localizing to different organelles, not only for HeLEA1 homologs (Fig. 1C–E) but for other LEA protein families, as reported in *Arabidopsis* (26). Such a biophysical mechanism may represent a simple and evolvable solution that can confer stress tolerance across the different domains of life (Fig. 7C). The subcellular localization, disordered state conformation, and biophysical properties of the structured state, may work synergistically in the divergence of protein sequence but conservation of molecular function for LEA proteins, and probably for other families of IDPs during evolution.

## Materials and methods

### Sequence search and bioinformatics

An iterative sequence search was performed with HMMER (16), which identified four proteins that exhibited positive fitness under stress when heterologously expressed as queries (UniProt ID: P0CU49, P0CU50, P0CU51, and P0CU52), with a low cutoff of 1E−2. One homolog containing unidentified amino acid residues was removed from the analysis. All-against-all sequence similarity analysis and sequence clustering were performed with EFI tools (17). The network was visualized with Cytoscape. The structural homology search was performed with PhyRE2 (40).

Subcellular localization predictions were performed primarily by using WoLF-PSORT (84), and the highest-ranked localizations were reported. TargetP 2.0 (85) was also used to validate the WoLF-PSORT predictions. Both algorithms agreed reasonably well with each other (84 of 96 matched, Table S4). The prediction results of WoLF-PSORT were used when a conflict occurred between the two algorithms. The cleavage sites of each targeting signal were predicted with TargetP 2.0. The targeting signal–cleaved sequences were used as the input for another multiple sequence alignment with the MAFFT method in the online tool Wasabi (86, 87). The phylogenetic tree was created based on this alignment by using MEGAX with the maximum likelihood method and WAG (*g* + *i*) substitution method (88). Disorder and charge pattern analyses were performed by using an in-house script that integrated methods from localCIDER (89) with a sliding window size of 10 and IUPred2A (90) with the long disorder mode.

The amphipathic helices predictions were performed with an in-house script. The general flow of the algorithm is illustrated in Fig. S11. Briefly, an input sequence is entered into the algorithm, which breaks it into subsequences when any of the helix-break criteria were satisfied: proline, continuous glycines or over four (including four) continuous polar/negative residues. The subsequences were then broken into tiles with a sliding window of seven residues according to previously reported criteria for the length of valid amphipathic helices (43). Amphipathicity was verified for each subsequence of seven residues according to the criteria for apolipoprotein structural motifs (44). Valid adjacent amphipathic subsequences of seven residues were stitched together to form the final output. AlphaFold2 prediction of HeLEA1 was downloaded from AlphaFoldDB (91).

To identify repeating motifs in HeLEA1 homologs, we used 96 HeLEA1 homolog sequences after predicted processing of signal peptides as the sequence inputs for motif discovery with MEME (22), with an upper bound motif length of 11 and -anr option. The upper bound motif length 11 was chosen as the repeating unit of 3–11 helices. For each occurrence of a motif in each sequence, a cutoff of 1E−4 was used as the significance cutoff.

Analysis of the biophysical properties of the identified helices in HeLEA1 homologs was performed by using a custom-written script according to Fig. S18. Briefly, each input homolog sequence was subjected to (i) IUPred2A (90) disorder prediction and (ii) 3–11 helix identification according to Fig. S11. Four parameters were calculated for the residues in each identified helix: (i) mean IUPred score; (ii) the mean hydrophobicity (H); (iii) hydrophobic moments (µH); (iv) discriminant factor *D* = 0.944 × *µH* + 0.33 × *z*, where *z* is the total charge carried by each element. The latter three properties were calculated using the hydrophobicity parameter and equations from HELIQUEST (42). The median differences between the compared groups, common language effect size and *P*-values were calculated with the *R* package *coin*.

Analysis of the biophysical properties of the identified helices in HeLEA1 homologs with respect to different localizations was performed similarly as described above. The elements were divided into four groups: mitochondrial, chloroplast, secretory, and others, based on predictions from TargetP. A Kruskal–Wallis rank sum test was used for comparing the properties of multiple groups.

### Strains and plasmids

The yeast strains and plasmids used in this study are listed in Table S5. Standard cloning methods were used, including PCR amplification of tardigrade genes and overlap extension with the Phusion-HF enzyme (Agilent) as per the manufacturer’s instructions. For the strains used in functional assays, the yeast endogenous *CAN1* locus was used to insert an expression cassette consisting of either *HeLEA1_FL_* or *HeLEA1 (39–238)* under a constitutive *TDH3* promoter and *ADH1* terminator. Tom20p was tagged with EGFP by using homologous recombination and the *EGFP-HIS3MX6* cassette (92).

### Cell imaging

Yeast strains with *TOM20* genomically tagged with *RFP* (63) were transformed with plasmids containing *GFP* fusions of *HeLEA1_FL_* or *HeLEA1*. The cells were photographed with a 100× 1.49 NA objective on a Nikon TI2 epifluorescence microscope with an sCMOS camera (Andor) and RFP and GFP filters (Chroma Technology, Rockingham, UT, USA). Images were processed with ImageJ.

### Immunoblot

Yeast cells expressing either *HeLEA1_FL_-HA* or *HeLEA1-HA* under a *TDH3* promoter were grown until an OD_600_ of 0.6–0.8 for log-phase samples, and 4–7 for stationary-phase samples, before collection. Cells from 2-mL culture samples were collected and treated with 0.2 M NaOH after washing with water and boiling with loading buffer (0.187 M Tris pH 6.8, 30% [v/v] glycerol, 6% [w/v] SDS, 0.1% [w/v] bromophenol-blue and 0.2 M dithiothreitol [DTT]). The cell lysates were separated on 4–12% NuPAGE Bis–Tris gels and immunoblotted with a mouse anti-HA antibody as primary diluted 1:10,000 and HRP-conjugated goat antimouse secondary antibody diluted 1:10,000. Erv46p was used as western blot loading control (93).

### Protein expression, purification, and labeling

#### Protein expression

For regular expression, a plasmid containing His-tagged *HeLEA1 (39–238)* gene was transformed into BL21(DE3) cells (New England Biolabs). Overnight cultures in 1 mL of LB media were used to inoculate 1 L of LB media split into two 500-mL cultures, each in a 2-L baffled flask. The cells were grown at 37 °C to late log-phase (OD_600_ ∼1.0–1.2) before induction with 1 mM IPTG. Cells were harvested at 3.5–4 h postinduction and stored at –80 °C until purification.

#### Protein purification

The cell pellets were resuspended in lysis buffer (50 mM Tris pH 8.0, 500 mM NaCl, 10 mM imidazole, 1 mg/mL lysozyme, one tablet of EDTA-free complete protease inhibitor [Roche] and 1 mM PMSF) before ultrasonication to lyse the cells. The mixture was then centrifuged at 20,000 × *g* for 30 min to remove cell debris. The supernatants were incubated with 5 mL of Ni NTA-resin (QIAGEN) for 1 h at 4 °C before elution with high imidazole buffer. To remove the His-tag, Tobbaco Etch Virus (TEV) protease and 1 mM DTT were added to the eluates, and the mixtures were dialyzed against a low imidazole buffer. The samples were then loaded onto a second NTA-column to remove the cleaved His-tag. The unbound flow- through containing the purified protein was buffer exchanged into the size-exclusion chromatography buffer (pH 6.5, 50 mM K^+^ phosphate, 100 mM NaCl, and 1 mM DTT). The protein was then further purified with a Superdex 200 column (GE Healthcare), and the purity was verified by SDS–PAGE. Pure fractions were pooled, concentrated, aliquoted, snap-frozen in liquid nitrogen, and stored at −80 °C until use. Ten percent of TFE samples used for NMR and SAXS experiments were prepared by diluting stock protein aliquots with size-exclusion chromatography buffer and 2,2,2-TFE (Fisher Scientific), to make the final concentration of TFE 10%.

#### Isotopic labeling

Isotopically labeled proteins were overexpressed in M9 media (6 g/L Na_2_HPO_4_, 3 g/L KH_2_PO_4_, and 0.5 g/L NaCl) supplemented with 1.7 g/L yeast nitrogen base without NH_4_Cl and amino acids (Sigma). In addition, 1 g/L ^15^NH_4_Cl and 4-g/L ^13^C glucose (Sigma) were supplemented for ^15^N and ^13^C labeling.

#### Fluorescence labeling with Alexa-488

Pure HeLEA1 (39–238) protein was labeled at the N-terminus with Alexa-488 succinimidyl ester (Thermo Fisher) as previously reported (94). Briefly, the purified protein was buffer exchanged into 100 mM sodium bicarbonate buffer (pH 8.5) and concentrated from ∼200 µL to around 250 µM. One aliquot of dye freshly dissolved in 10 µL of anhydrous DMSO was mixed with the protein and incubated at room temperature for 30 min. This was followed by further incubation overnight at 4 °C with protection from light. The labeling mixture was purified by using coupled 1.5-mL HiTrap desalting columns (GE Lifescience), snap frozen, and stored at −80 °C until use.

### NMR spectroscopy

Experiments were performed at 278 K with a Bruker AvanceIII 800 MHz spectrometer equipped with a TCI CryoProbe, including 50 µM samples in 50 mM phosphate buffer and 100 mM NaCl at pH 6.5. Lipid binding experiments were acquired in the same buffer at pH 7.4.

Backbone amide resonances were obtained with the following triple resonance 3D experiments (standard Bruker pulse sequence library): HNCO, HN(CA)CO, HNCA, HNCACB, HN(CO)CACB and HN(COCA)NNH. HBHA(CO)NH and ^15^N-NOESY-HSQC experiments enabled the assignment of Hα resonances. All 3D datasets were collected with nonuniform sampling at 20–50% and processed with compressed sensing in MddNMR (95) (Swedish NMR Centre) and NMRPipe. Experiments with ^13^C-detected CON, (HACA)CON, and (HACA)NCO (Bruker pulse sequence library) were used to confirm amide-based assignments in crowded spectral regions. Topspin 3.6 (Bruker), NMRFAM-Sparky 1.47 (96), and Mars (97) were used for processing, data analysis, and backbone assignment, respectively.

#### Secondary chemical shifts

*Cα/Cβ* chemical shift deviations were calculated with the following equation:


$$\Delta \delta C_{\alpha \beta }=(\delta C\alpha_{\mathrm{obs}}\;\text{–}\;\delta C\alpha_{\mathrm{rc}})\;\text{–}\;(\delta C\beta_{\mathrm{obs}}\;\text{–}\;\delta C\beta_{\mathrm{rc}})\;[\mathrm{ppm}],$$

where *δCα*_obs_ and *δCβ*_obs_ are the observed *Cα* and *Cβ* chemical shifts, and *δCα*_rc_ and *δCβ*_rc_ are *Cα* and *Cβ* chemical shift values for residues in random coils (98) compensated via temperature coefficients (99) and correction factors for side chain per deuteration (100).

#### ^15^N-relaxation measurements

The ^15^N *T*_2_ (1/*R*_2_) and *T*_1_ (1/*R*_1_) relaxation times were measured by using standard HSQC-based pseudo-3D pulse sequences (Bruker) with recycle delays of 5 s. *T*_2_ datasets were acquired with 16 CPMG delays between 8.5 and 271 ms and *T*_1_ datasets consisted of 11 recovery delays from 10 ms to 2 s. Peak heights were analyzed in NMRFAM-Sparky 1.47 (96) and exponential decay rates (*R*_2_ and *R*_1_) were fitted according to *h* = *A* × exp(−*R* × *t*), where *h* is the observed peak at a given relaxation time *t*. The ^15^N{^1^H}-hetNOE measurements were performed with standard 2D Bruker pulse sequences, with interscan recovery delays of 5 s and interleaved on-resonance (*I*) or off-resonance (*I*_0_) saturation. The ^15^N hetNOE values are expressed as the *I*/*I*_0_ ratio.

### Small-angle X-ray scattering measurements

In-line size-exclusion chromatography SAXS data of HeLEA1 (39–238) were collected at the B21 diamond light source with an Agilent 1200 HPLC and 2.4-mL Superdex S200 column (GE Healthcare). A 50 μL solution of HeLEA1 (39–238) at 5 mg/mL (∼240 μM) was loaded onto the S200 column in running buffer (50 mM phosphate-K^+^ and 100 mM NaCl) at pH 6.5. Frames were collected at 3 s per frame at 25 °C and X-ray scattering was recorded (Pilatus 2M detector) at a fixed camera length of 4.014 m, at 12.4 keV. Angular *q-*range data were collected between 0.0025 and 0.34 Å^–1^. Data reduction and buffer subtraction were performed with ScÅtter 3.1r (101). The resulting files were used as input for either ASTEROIDS (51) simulations or fitted with SAXSonIDP (102) for inference of *R_g_* distribution.

### ASTEROIDS simulation and ensemble analysis

A statistical coil ensemble of HeLEA1 (39–238) comprising 10,000 conformers was generated with *flexible-meccano* (52). Two hundred conformations that best described the experimentally obtained backbone N, HN, Cα, Cβ, and CO chemical shifts were selected from the ensemble by using the genetic algorithm *ASTEROIDS* (51). A new ensemble of 8,500 conformers was generated according to the phi and psi angles of the selected conformers. These conformers were mixed with 1,500 conformers from the initial statistical coil ensemble (51, 52) to create a combined ensemble in the next iteration. This new ensemble was subjected to another round of ASTEROIDS selection, and the iteration step was repeated nine times until the ensemble converged with respect to the chemical shifts. Ensemble-averaged chemical shifts were calculated with SPARTA (103).

For the integration of SAXS data, an ensemble of 100,000 conformers was generated according to the phi and psi angles of the ensemble selected based on the chemical shifts. ASTEROIDS were then used for the selection of 100 conformers according to the restrictions of both chemical shifts and the SAXS curves. Ensemble-averaged SAXS curves were obtained with CRYSOL (104).

### Liposome generation

The following lipids were used in this study (with abbreviations): 1,2-dimyristoyl-sn-glycero-3-phospho-l-serine (sodium salt, DMPS), 1-palmitoyl-2-oleoyl-sn-glycero-3-phospho-l-serine sodium salt (sodium salt, POPS), 1-palmitoyl-2-oleoyl-glycero-3-phosphocholine (POPC), 1-palmitoyl-2-oleoyl-sn-glycero-3-phosphoethanolamine (POPE), l-α-phosphatidylinositol (from the bovine liver) (sodium salt, PI), cardiolipin (from the bovine heart) (sodium salt), and 1,2-dioleoyl-sn-glycero-3-phosphoethanolamine-*N*-(Lissamine rhodamine B sulfonyl) (ammonium salt) (DOPE-lissamine rhodamine) were purchased from Avanti Polar Lipids (Alabaster, AL, USA).

The SUVs consisting of POPS, DMPS, or POPC and POPE, used for CD, NMR, and DSC experiments were generated by using the ultrasonication method. Briefly, lipid stock solutions in chloroform were measured by using a Hamilton syringe to transfer the samples into glass tubes. The solution was allowed to dry in a nitrogen flow and vacuumed overnight under the protection of light to remove residual solvent. The following day, lipids were rehydrated at room temperature (for POPS with *T_m_* at 14 °C) or 42 °C (for DMPS with *T_m_* at 35 °C) for at least 1 h before ultrasonication with a microprobe until the solution turned clear. Dynamic light scattering using a DynaPro PlateReader-II (Wyatt) was used to establish that the size of the liposomes was <70 nm.

The SUVs comprising POPS or POPC used for the liposome floatation assays were generated by using the extrusion method. We added 2% of DOPE-lissamine rhodamine to the chloroform lipid stock solution, which was subsequently dried and vacuumed. After rehydration at room temperature, the lipid suspension was extruded 17 times through a polycarbonate filter with a 50-nm pore size. The size of the liposomes was measured by dynamic light scattering to confirm that the radii of the liposomes were <100 nm.

The IMM-composition mixture contained the following components: 40% POPC, 25% POPE, 10% liver PI, 5% POPS, 15% heart CA, and 0.5% DOPE-lissamine rhodamine based on previous report (65). For making IMM-composition SUVs, we used the same extrusion method as described above. For generating IMM-composition GUVs, we used the electroformation method as described below. A 40 μL solution of 1 mM lipid mixture in chloroform:methanol (95:5) was spread layer by layer on a circular region identified by a rubber O-ring of an indium-tin-oxide (ITO)-coated glass slide. The ITO-coated slide was vacuumed overnight and protected from light to remove any solvent. On the second day, two ITO-coated slides were coupled with 0.8-mm thick rubber spacers to form a chamber. A 600-µL rehydration solution (20 mM HEPES and 295 mM sucrose) with 300 nM HeLEA1 (39–238) labeled with Alexa-488 was added to rehydrate the lipid layers. The assembled ITO-coated slides were placed in a Vesicle Prep Pro electroformation chamber (Nanion) with an applied electric field of 10 and 1 V amplitude at 60 °C for 65 min.

### Liposome floatation assay

Liposome-binding experiments were performed with a modified protocol, as previously described (105). Briefly, 1 mM SUVs generated via extrusion (with 2% of DOPE-lissamine rhodamine) were mixed with 1 µM HeLEA1-Alexa-488 in 75 µL of binding buffer (20 mM HEPES with 50–500 mM NaCl pH 7.4) and incubated at room temperature away from light for 30 min. Each sample was mixed with 50 μL of 2.5 M sucrose and binding buffer. A 100 µL aliquot of the mixture was then transferred to an ultracentrifuge tube, overlaid with 100 μL of 0.75 M sucrose and binding buffer and 20 μL of binding buffer. The gradients were centrifuged (100,000 rpm) for 90 min at 20 °C with slow acceleration/deceleration in an ultracentrifuge with a Beckman TLA-100 rotor. The top 40 μL of the gradients were collected and normalized for lipid recovery by using the absorption of the rhodamine dye at 582 nm. Samples were then resolved by SDS–PAGE and visualized with an Amersham Typhoon gel imager observing the fluorescence from HeLEA1-Alexa-488.

### Circular dichroism spectroscopy

CD spectra were collected on a JASCO J-815 CD spectrometer from 260 to 200 nm with 0.5-nm intervals at 20 °C. For each spectrum, 10μM HeLEA1 protein was diluted in CD buffer (10 mM Phosphate-K^+^ and 20 mM NaCl pH 7.5) and placed in a 0.1-cm quartz cuvette. The spectra were obtained at a 20 nm/min scanning speed with standard (100 mdeg) sensitivity and averaged three times. For free protein, a blank buffer trace was subtracted to establish the background. For titration with liposomes at each concentration of liposome, a corresponding blank trace without protein was measured and subtracted for correction. For titration with different fractions of TFE, the stock protein was diluted to a final concentration of 10 μM in CD buffer containing various fractions of TFE. The processed data were then plotted using Graphpad Prism 8.0. The thermal melting of HeLEA1 bound to POPS liposomes was performed using 10 μM of HeLEA1 with 2 mM POPS SUV to ensure the binding of HeLEA1 to SUVs was saturated. Scanning was performed from 5 to 70 °C with a scanning speed of 10 °C/h. After the cooling experiment, the sample was measured by dynamic light scattering (DLS) to confirm that the SUVs still stably existed in solution. Deconvolution of the CD spectra to reveal an increased fraction of secondary structures was performed with BeStSel (62). The full titration curve with saturation point was used as input.

### Differential scanning calorimetry

DSC measurements were performed using a MicroCal VP-capillary DSC system (GE Healthcare). 2 mM SUVs made of 100% POPS, 100% DMPS, or 1:1 POPC:POPE, in measurement buffer (20 mM HEPES, 150 mM NaCl, pH 7.4) were mixed with 10 µM HeLEA1 protein or identical volume of buffer alone before DSC measurements. DSC thermograms were determined by monitoring the difference in heat capacity in solution upon the increasing temperature at a scan rate of 10 °C/h. A parallel of at least three samples for each entry was measured and the thermograms agreed well within repeats. Buffer traces without any lipid or protein were used for buffer subtraction and establishment of the baseline. The data were analyzed using software associated with the system to perform buffer subtraction. The final baseline was corrected using the average of the last 15 points of measurements, well beyond the phase transition of the liposome.

### Preparation of Langmuir monolayers and measurement of isotherms

Surface pressure–mean molecular area (π–A) isotherms were performed on a 364 × 74 mm^2^ KSV trough (Biolin Scientific, Gothenburg, Sweden). Barrier control and data acquisition were achieved by using KSV NIMA software. Surface pressure was recorded by a prewetted paper Wilhelmy plate connected to a microelectronic system during compression at 22 ± 1 °C. To measure the isotherms of lipids, the subphase comprised 50 mM phosphate buffer and 100 mM NaCl at pH 7.4. To measure the influence of HeLEA1 on the lipid monolayer isotherms, the subphase comprised phosphate buffer (50 mM phosphate buffer and 100 mM NaCl at pH 7.4) containing 3 nM HeLEA1. Before the subphase addition, the trough was cleaned twice with ethanol and once with Milli-Q water. The lipids were dissolved in chloroform at a concentration of 1.3 mg/mL. The lipid solutions were added drop by drop and spread onto the subphase with a high-precision Hamilton microsyringe. The solvent was allowed to evaporate for 15 min before lipid monolayers were compressed. The compression rate was 10 mm/min. The isotherms were obtained by measuring the surface pressure as a function of the molecular area during lipid monolayer compression. The elastic compressibility can be determined from the surface pressure versus mean molecular area isotherm by calculating the corresponding slope:


$$C_{s}^{\left(-1\right)}=-A\left(\frac{d\pi }{dA}\right),$$

where $C_{s}^{-1}$ is the compression modulus, *A* is the mean molecular area, and *π* is the corresponding surface pressure.

### GUV imaging

GUVs with encapsulated HeLEA1-Alexa488 were imaged with an inverted Zeiss 710 confocal microscope with 63×/1.4NA oil objective. The GUVs were placed in an eight-well μ-slide (Ibidi) coated with 1-mg/mL casein solution to prevent any nonspecific sticking. The GUVs generated via electroformation in rehydration buffer (20 mM HEPES pH 7.4 and 295 mM sucrose) were diluted with an equal amount of 300 mM glucose and HEPES buffer (20 mM HEPES pH 7.4 and 150 mM NaCl). The images were collected with ZEN software (Zeiss) and analyzed by ImageJ.

### Yeast serial dilution assay

For serial dilution assays, yeast strains were grown to saturation in YEP + 2% glucose (YEPD) medium overnight at 30 °C. Five-fold serial dilutions were made in 96-well trays before spotting 2 µL onto corresponding plates with different stresses and carbon sources. Plates were incubated at 37 °C and scanned 2 or 3 days after spotting.

### Flow cytometry

Yeast cells were grown at 30 °C overnight in YEPD media. On the second day, cells were diluted in either YEPD or YEP + 3% glycerol (YEPG) media until the OD_600_ reached 0.4–0.6. The cells were stained with 20 nM tetramethylrhodamine, methyl ester (TMRM) at 30 °C for 30 min before analysis with a Becton Dickinson LSRII analyzer. For each experiment, 60,000 cells were analyzed. The data were further processed and analyzed with FCS Express 7 software, applying standard gating on front and side scattering to eliminate dead cells and doublets. The median for each flow cytometry experiment for either the TMRM fluorescence channel or EGFP fluorescence channel was measured. The ratio $=\frac{F_{\mathrm{TMRM}}}{F_{\mathrm{EGFP}}}$ was used to quantify normalized mitochondrial membrane potential for relative comparison. Welch t tests were used to assess the statistical significance of changes in EGFP intensity or relative membrane potential.

### Purification of mitochondria

Mitochondria were purified by differential centrifugation method (106), with a modified spheroplasting protocol adapted from previous work (105). Briefly, yeast cells grown in either YEPD media until the OD_600_ reached 0.6 were harvested and subjected to spheroplasting with homemade lyticase. The spheroplasts were collected and resuspended in a homogenization buffer (10 mM Tris/HCl pH 7.4, 0.6 M sorbitol 1 mM EDTA 2 g/mL BSA) before being homogenized with a dounce homogenizer. The lysates were centrifuged at 1,500 × *g* for 5 min, and the supernatants were further centrifuged at 3,000 × *g* for 5 min, 12,000 × g for 15 min. The pellets from the second centrifugation were resuspended in homogenization buffer, centrifuged at 3,000 × *g* for 5 min, and 12,000 × *g* for 15 min. The final pellets were resuspended in SEM buffer (10 mM MOPS/KOH pH 7.2, 0.25 M sucrose, 1 mM EDTA), and concentrations of mitochondria were determined via Bradford assays. The mitochondria were flash-frozen and stored at −80 °C before use.

### Membrane fluidity measurements by DPH anisotropy

Temperature-dependent fluorescence anisotropy measurements were performed with a Cary Eclipse fluorimeter. The raw anisotropy for each measurement was calculated with the following equation and used for relative comparison:


$$\mathrm{Anisotropy}=\frac{I_{\left|\right|}-g\times I_{⊥}}{I_{\left|\right|}+2\times g\times I_{⊥}},$$

where $I_{\left|\right|}$ and $I_{⊥}$ represent the fluorescence intensity from the parallel and perpendicular channels, respectively; *g* represents the gamma factor to correct for detection difference between the detectors and was determined to be 1.171 using DPH in methanol as control. The SD of the repeats was used to generate error bars for each point. Two-tailed, unpaired t tests were used to assess the statistical significance at each temperature; a further two-way ANOVA test was performed for the whole temperature-dependent dataset to assess the statistical significance of the whole trace.

## Supplementary Material

pgae006_Supplementary_Data

## Data Availability

All study data are included in the article and/or supporting information.
