# Supplementary material for: Dynamic conformational changes of a tardigrade group-3 late embryogenesis abundant protein modulate membrane biophysical properties
Source: PNAS Nexus. 2024 Jan 18;3(1):pgae006. doi: 10.1093/pnasnexus/pgae006 (PMC10808001; doi:10.1093/pnasnexus/pgae006)
Supplement: pgae006_Supplementary_Data [file pgae006_supplementary_data.zip › PNASNEXUS-PNASNEXUS-2023-00701RR-s02.docx]

**Supporting Information for**

Dynamic conformational changes of a tardigrade group-3 late embryogenesis abundant protein modulate membrane biophysical properties

Xiao-Han Li^1^*, Conny W.H. Yu^1^, Natalia Gomez-Navarro^1^, Viktoriya Stancheva^1^, Hongni Zhu^2^, Andal Murthy^1^, Michael Wozny^1^, Ketan Malhotra^1^, Christopher M. Johnson^1^, Martin Blackledge^3^, Balaji Santhanam^1,4^, Wei Liu^5^, Jinqing Huang^2^, Stefan M.V. Freund^1^, Elizabeth A. Miller^1^*, M. Madan Babu^1,4^*

Corresponding authors: Xiao-Han Li, Elizabeth A. Miller, M. Madan Babu

Emails: [xli002@dundee.ac.uk](mailto:xli002@dundee.ac.uk), [emiller@mrc-lmb.cam.ac.uk](mailto:emiller@mrc-lmb.cam.ac.uk), [madan.babu@stjude.org](mailto:madan.babu@stjude.org)

**This PDF file includes:**

Supporting text

Figures S1 to S18

Tables S1 to S5

SI References


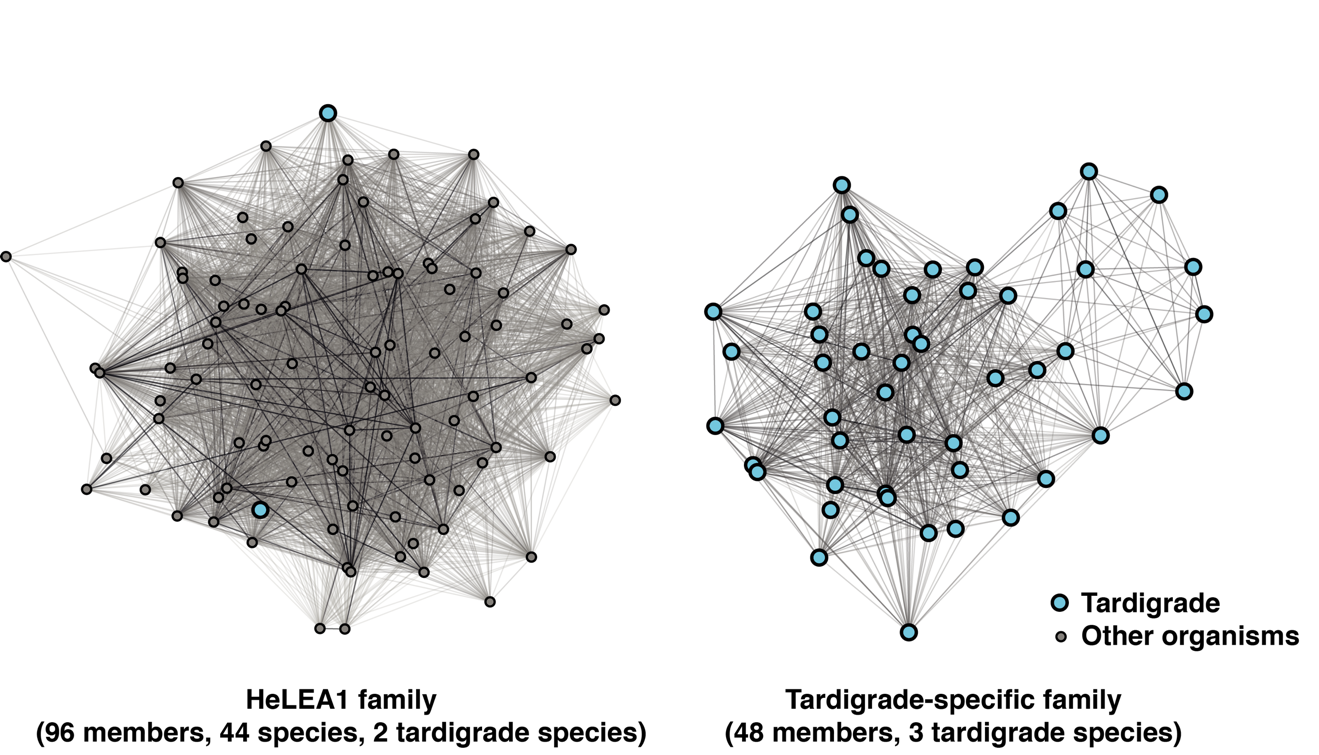


Fig. S1. A comprehensive sequence search identified two homologous protein families for tardigrade IDPs that confer desiccation tolerance when expressed in unicellular organisms. These include an evolutionarily conserved family (HeLEA1 family, left) and a tardigrade specific family (right). Homologs from tardigrades are highlighted in blue (Table S1 and Table S2). In this network, nodes represent individual sequences, and the edge weight represents the sequence identity over the aligned region.

**
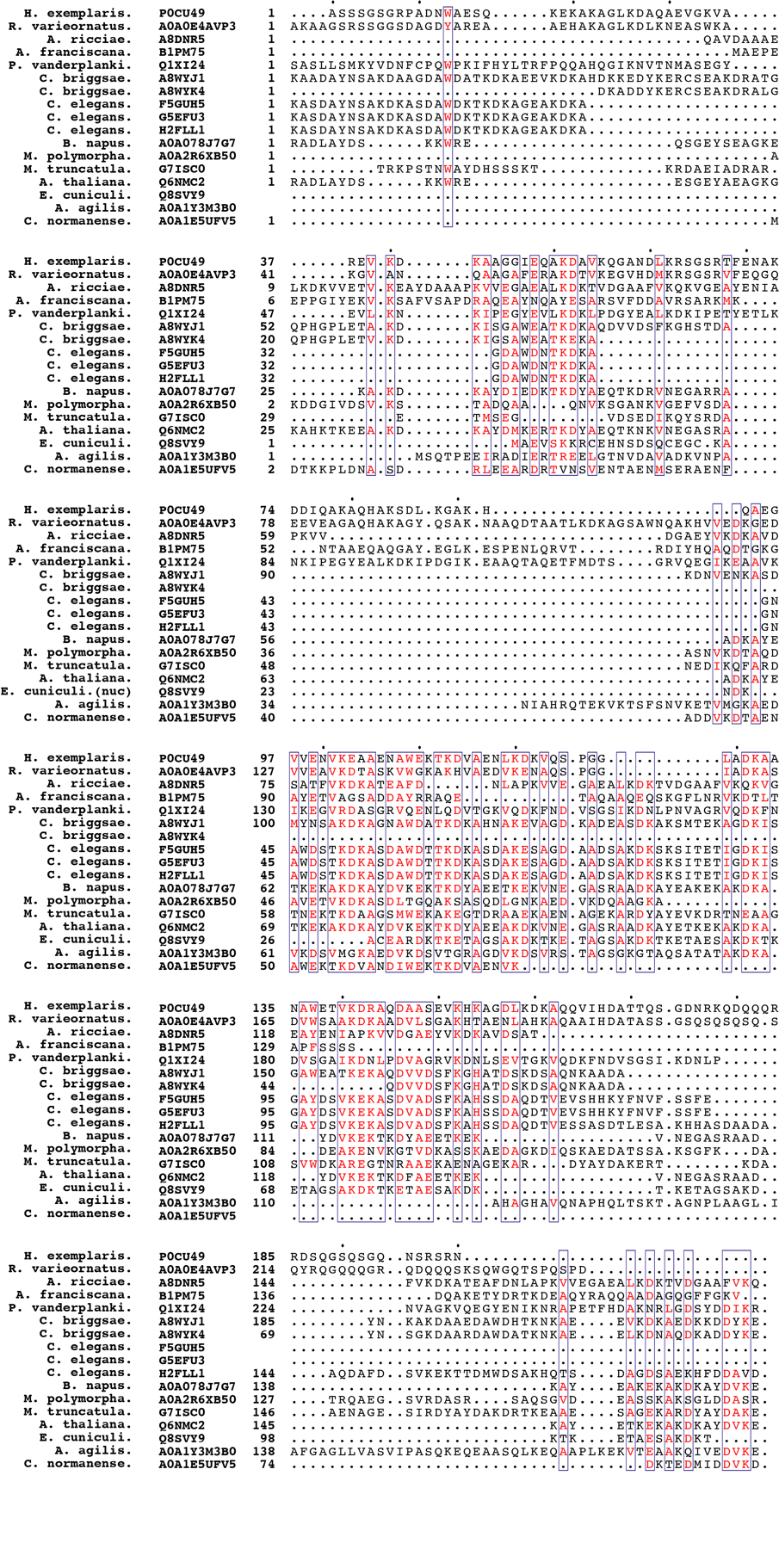
**

**Fig. S2 Sequence alignment of HeLEA1 and its representative homologs after removing predicted signal sequences.** For each sequence, the species of origin and UniProt ID are indicated. Alignment is visualized using ESPript, with %Equivalent coloring scheme, positions with global equivalent score over 0.5 are highlighted.


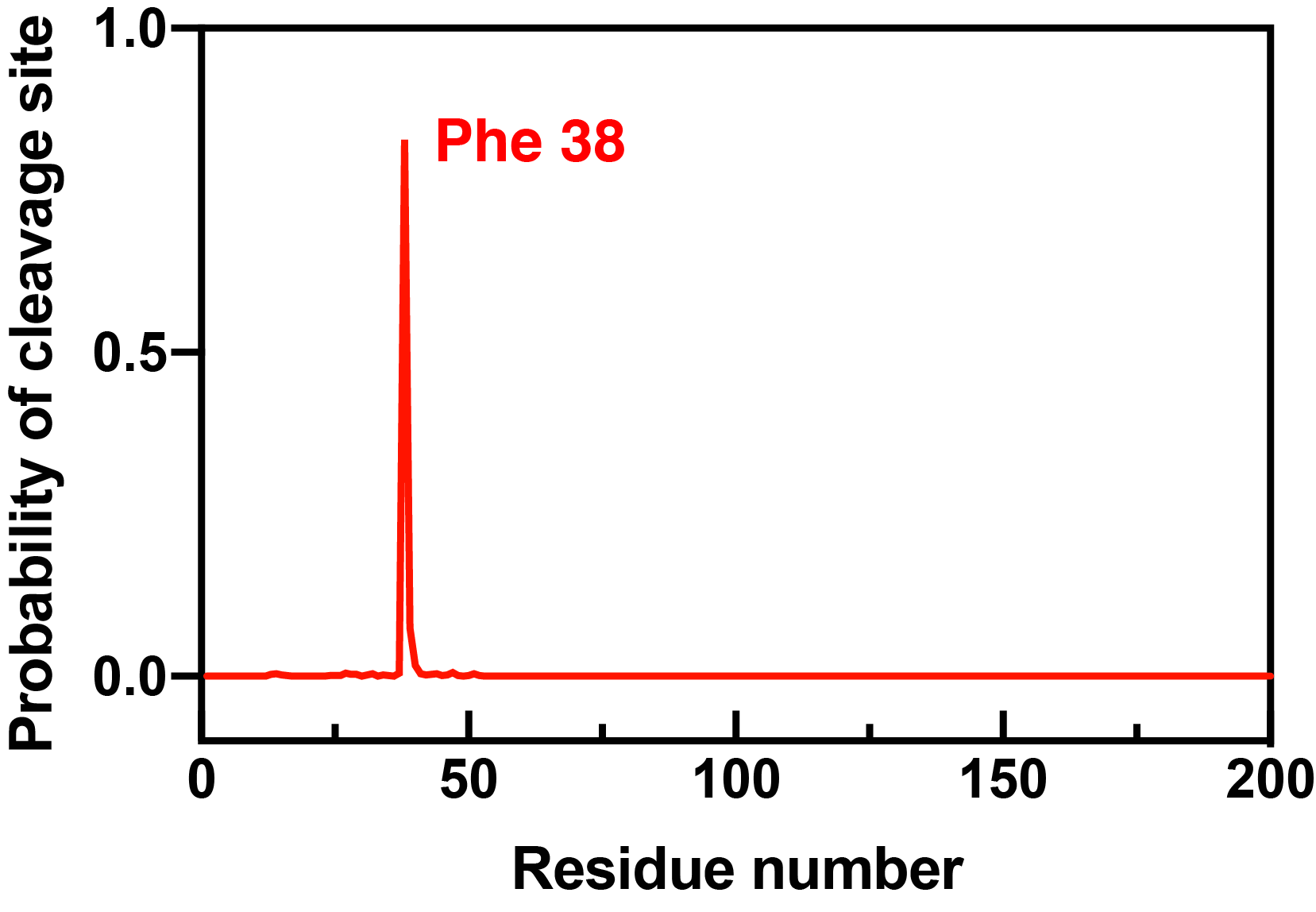


**Fig. S3 Prediction of location of the cleavage site of mitochondrial-targeting sequence by TargetP.** This suggests that full-length HeLEA1 has a high probability of being cleaved at phenylalanine 38 by the mitochondrial processing peptidase inside mitochondrial matrix.

**Fig. S4** The ^1^H_N_,^15^N 2D HSQC of HeLEA1 purified from *E. coli* shown with assignment of backbone resonances. Residues are colored according to their amino acid types.


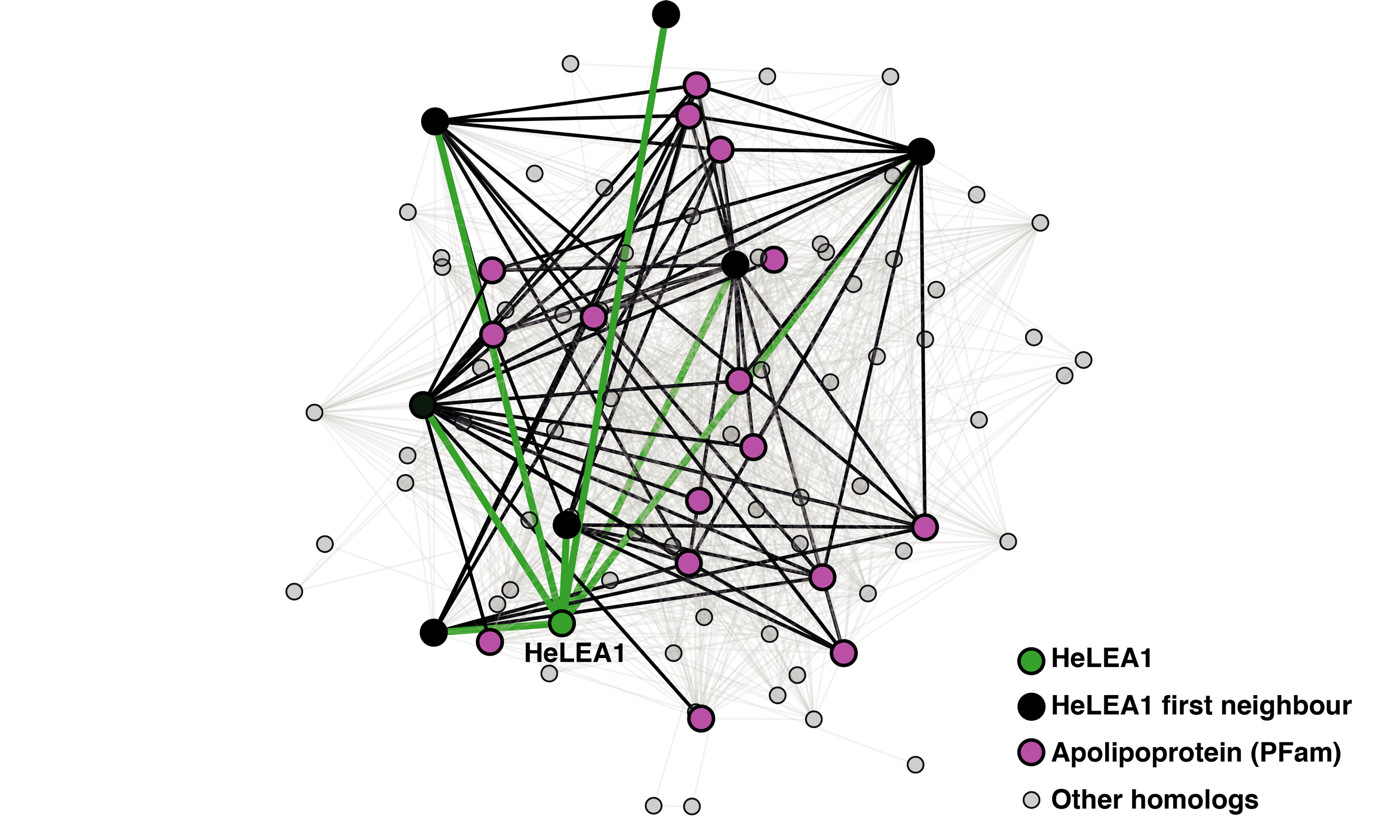


**Fig. S5** **HeLEA1 shares distant homology with annotated apolipoproteins.** Network constructed by pairwise sequence similarity was further filtered for pairs of homologs with > 40% identity. HeLEA1 (green) shares low similarity with an annotated apolipoprotein (magenta). However, six of the seven first neighbors of HeLEA1 (black) share a reasonable level of homology with apolipoproteins annotated in Pfam. The edges, which represent > 40% pairwise sequence identity in aligned regions, are highlighted in green (between HeLEA1 and its first neighbors) or black (between HeLEA1 first neighbors and annotated apolipoproteins).


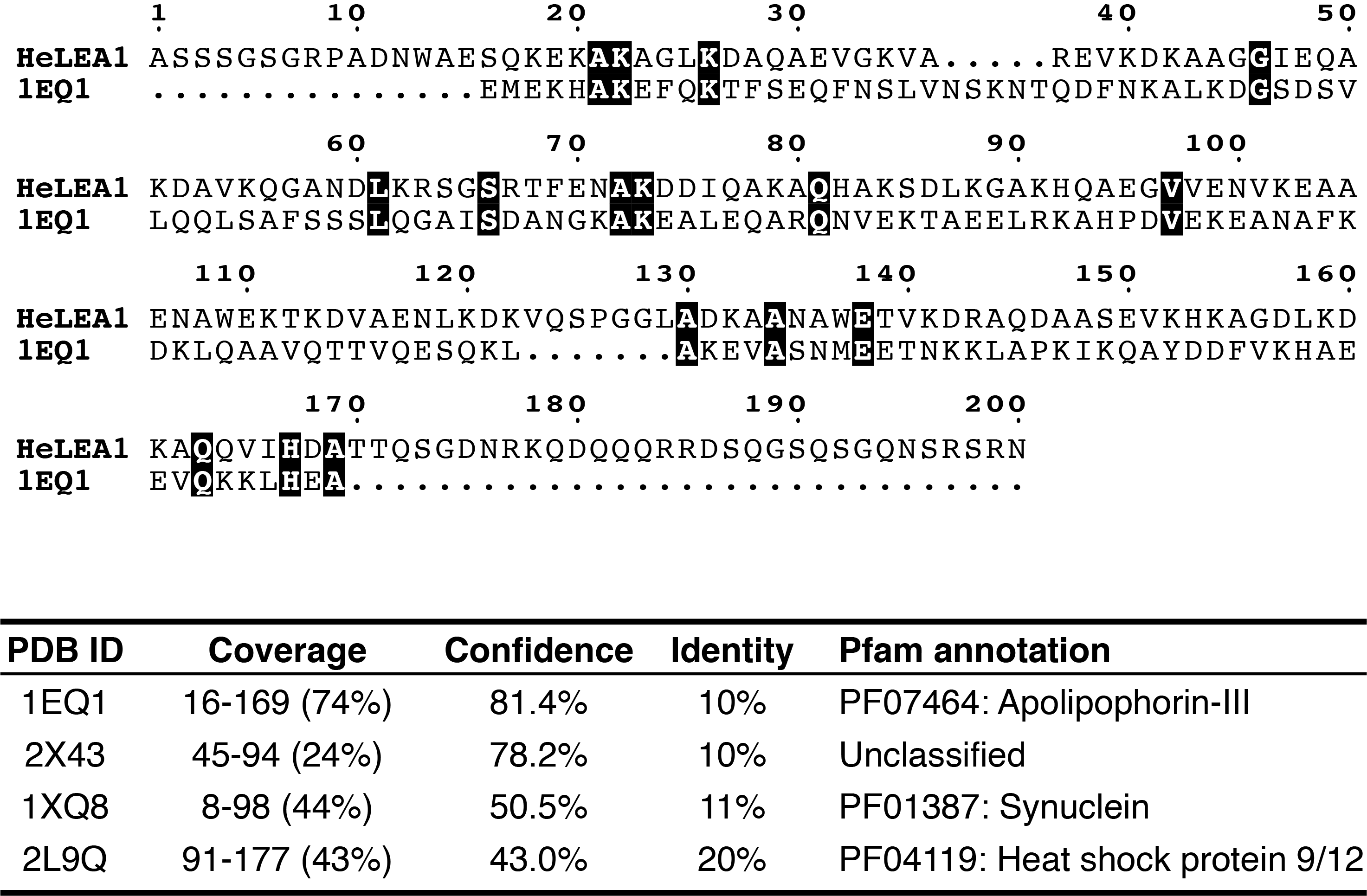


**Fig. S6** **HeLEA1 has weak sequence homology with lipid-interacting proteins.** **Top:** Sequence alignment between mature HeLEA1 and the top hit from a PhyRE2 homology search (PDB: 1EQ1), illustrating the very weak sequence similarity but good coverage of the sequence. **Bottom:** Summary of hits from the PhyRE2 search. Three of four hits (1EQ1, 1XQ8 and 2L9Q) contain the Pfam annotation of protein families involved in lipid interaction, although with low confidence to identify them as proper structural scaffolds for HeLEA1 (< 90%).


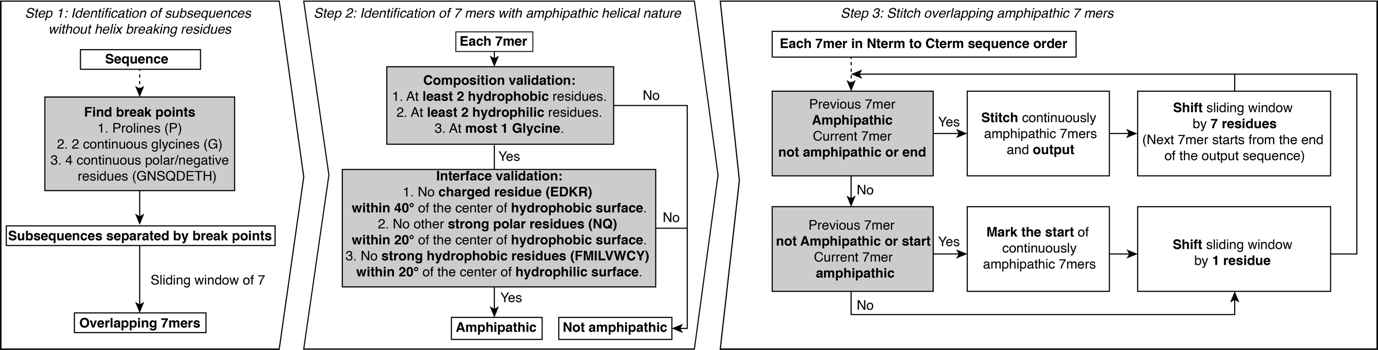


**Fig. S7** **Bioinformatics pipeline for discovery of amphipathic 3-11 helical motifs in a given protein, with a minimal length of 7.**


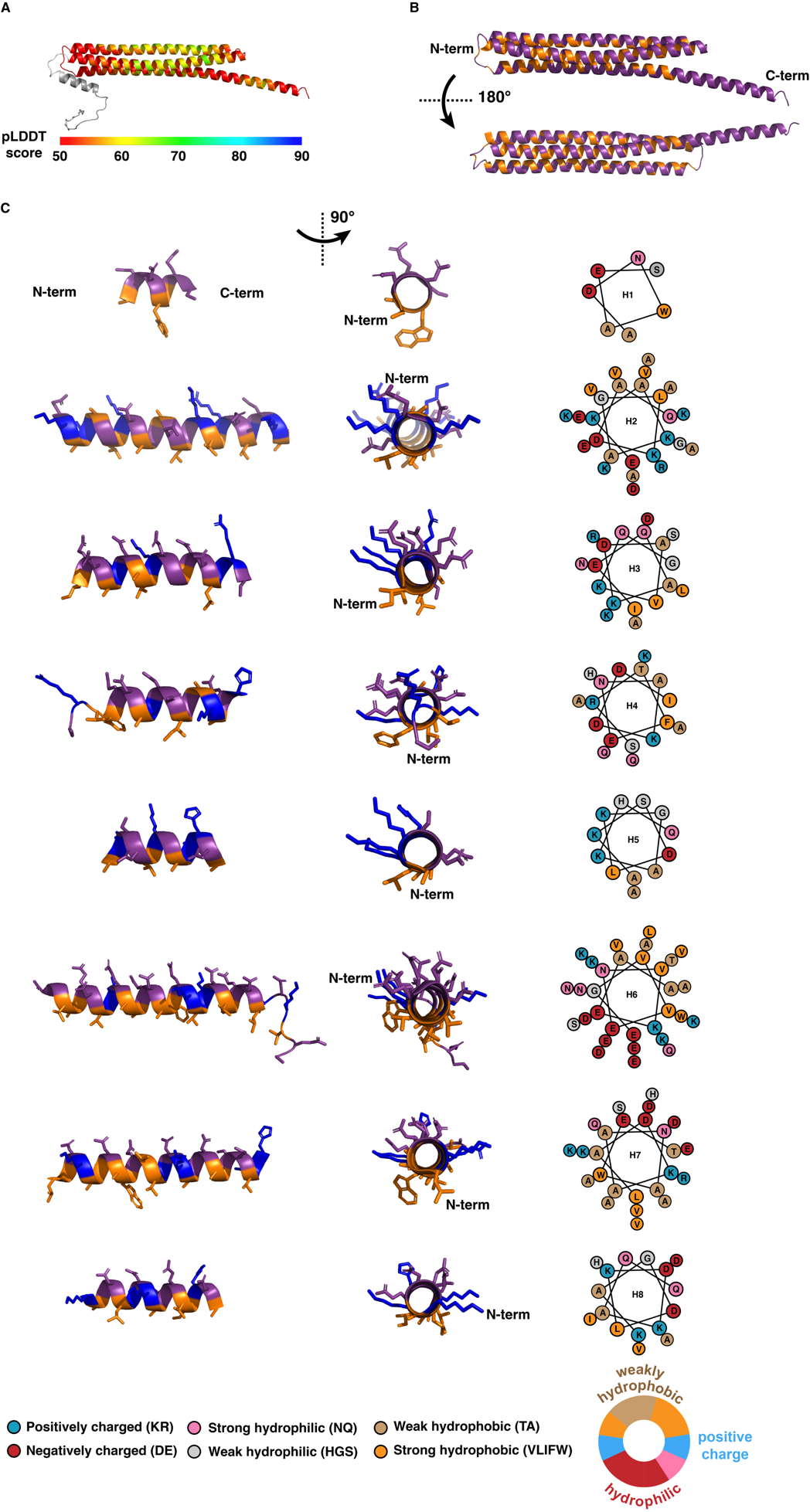


**Fig. S8 Predicted structure of HeLEA1. A,** AlphaFold2 prediction of HeLEA1_FL_, rendered by pLDDT score. The putative N-terminal MTS is rendered in gray. The low confidence score suggests the global higher-order structure is less reliable. **B,** AlphaFold2 prediction of HeLEA1, rendered by residue hydrophobicity. Hydrophobic residues (Ala, Cys, Phe, Ile, Leu, Met, Pro, Thr, Val, Trp, Tyr), are colored in orange, the rest of residues in purple. It is clear the AlphaFold predicted tertiary structure exhibits mismatching of amphipathic surfaces, and the tertiary structure is probably biased by the absence of membrane causing attraction between hydrophobic surfaces. **C,** Amphipathic elements predicted by biophysical criteria display coherent amphipathic surfaces.


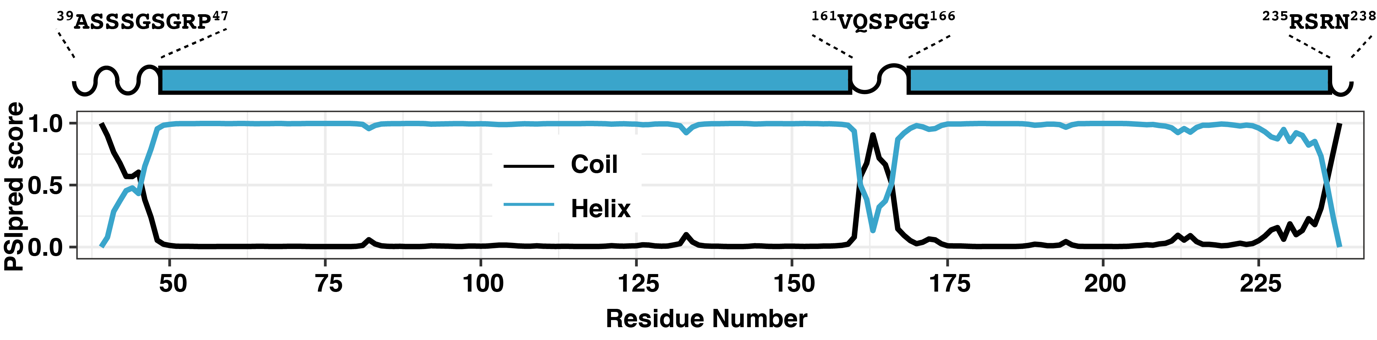


**Fig. S9** **PSIPRED data for secondary structure propensity in its functional form of HeLEA1.** Most of the residues in HeLEA1 are predicted to be helical with high confidence.


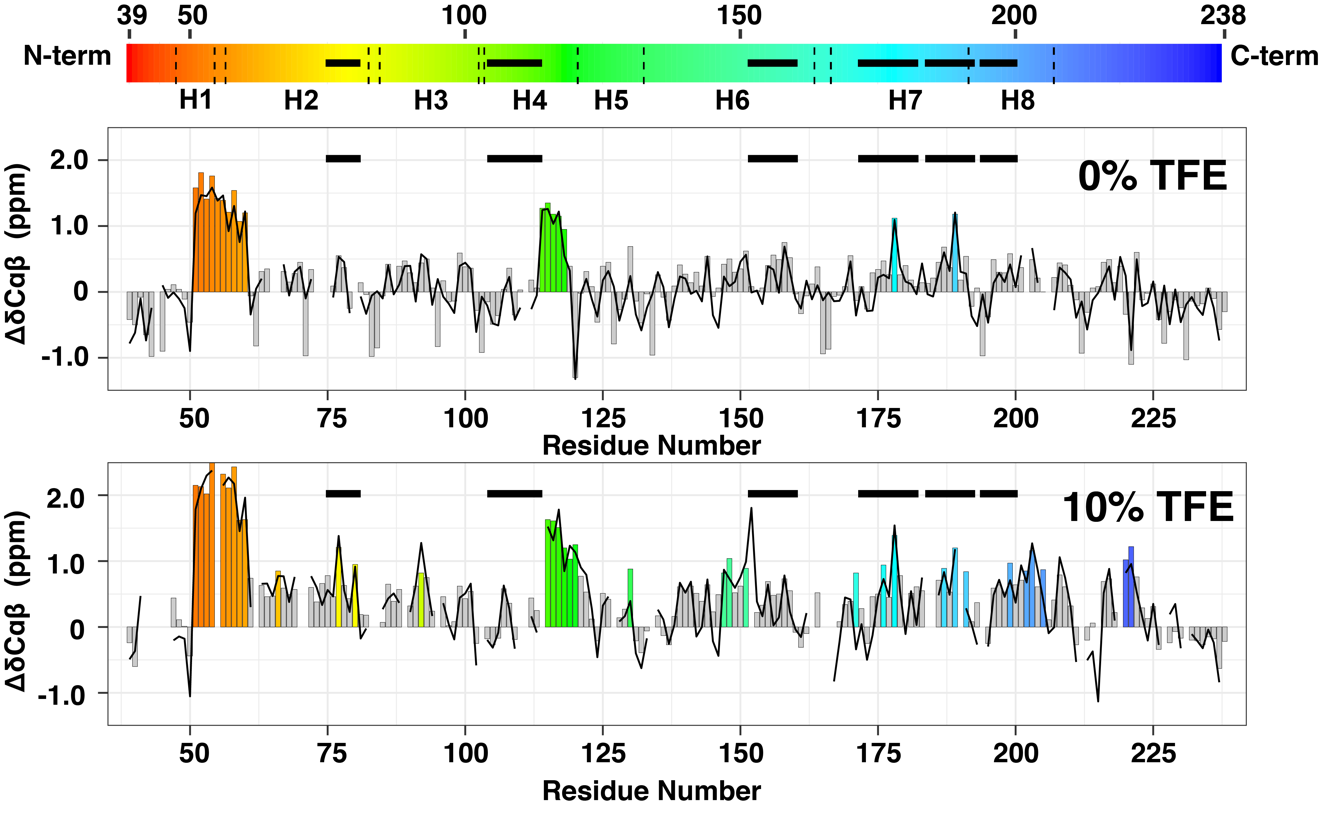


**Fig. S10 Secondary chemical shift (ΔδC_αβ_) of HeLEA1 in 0% TFE (top) or 10% TFE (bottom).** Black line represents *ASTEROIDS* fit of ΔδC_αβ_. Residues with significant positive secondary chemical shifts (> 0.8 ppm) are highlighted by color. Black bars represent conserved LEA motifs mapped in **Fig. 2A**.


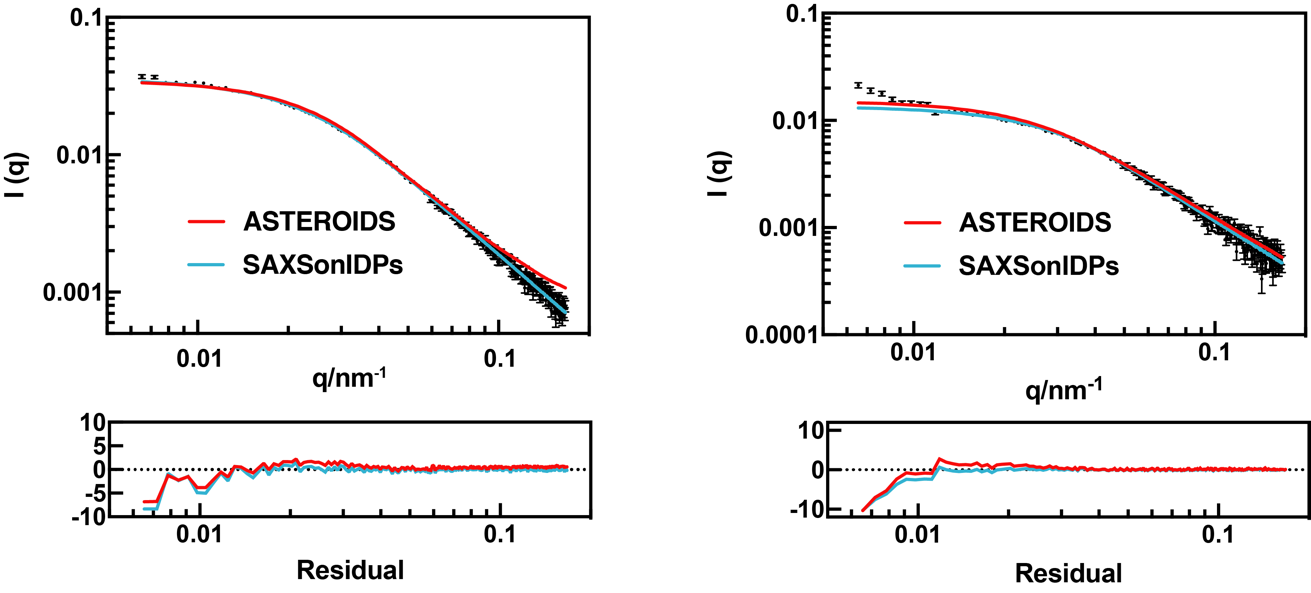


**Fig. S11 SAXS data and fitting of HeLEA1 in 0% TFE (left) and 10% TFE (right) using two different fitting methods.** The ensemble estimation by ASTEROIDS agreed well with the *SAXSonIDPs*(1) fitting. The residuals are standardized. SAXS data are represented as the mean ± SE (**Table S3**).


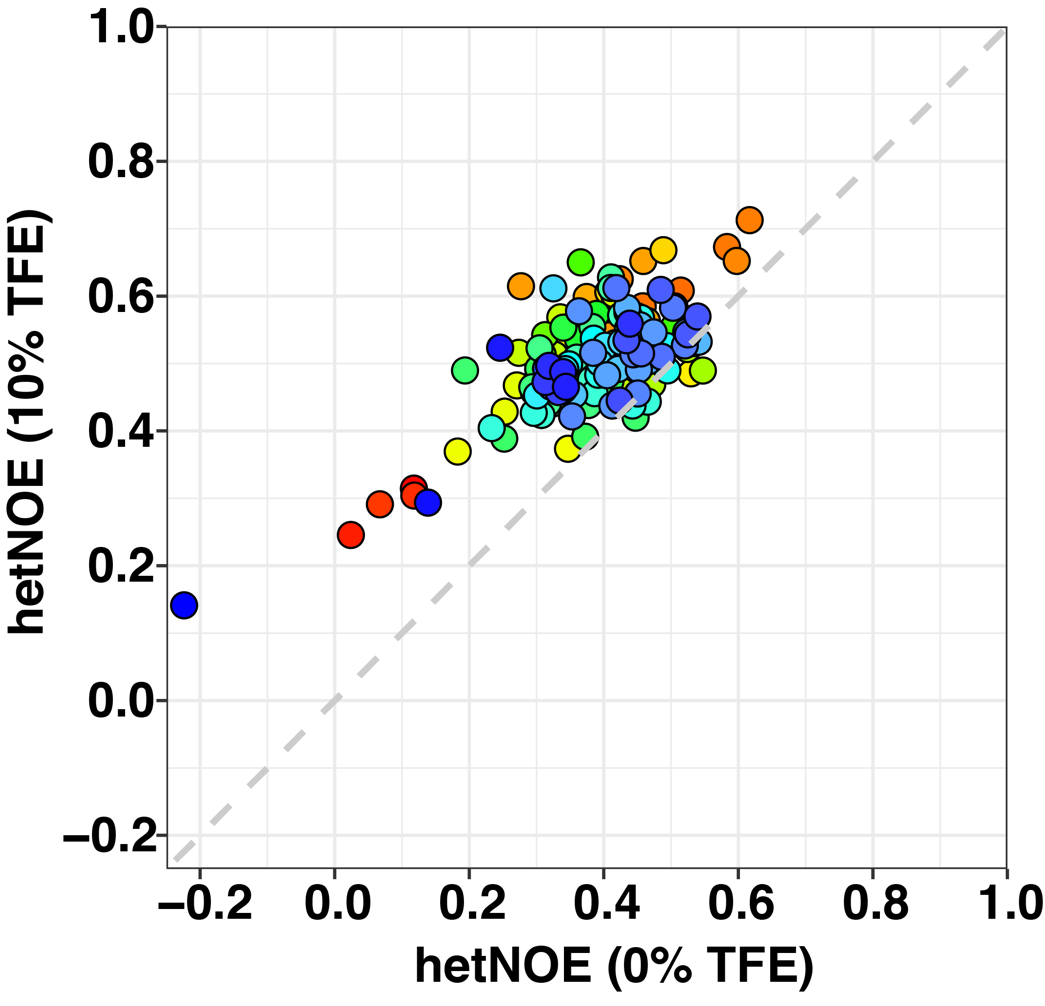


**Fig. S12** **Correlation of residue-specific ^15^N{^1^H} heteronuclear NOE (hetNOE) for HeLEA1 in 0% or 10% TFE.** A global increase of hetNOE were observed as a result of 10% TFE. The coloring scheme is the same as in **Fig. 2D, Fig. 2F,** and **Fig. S9**.


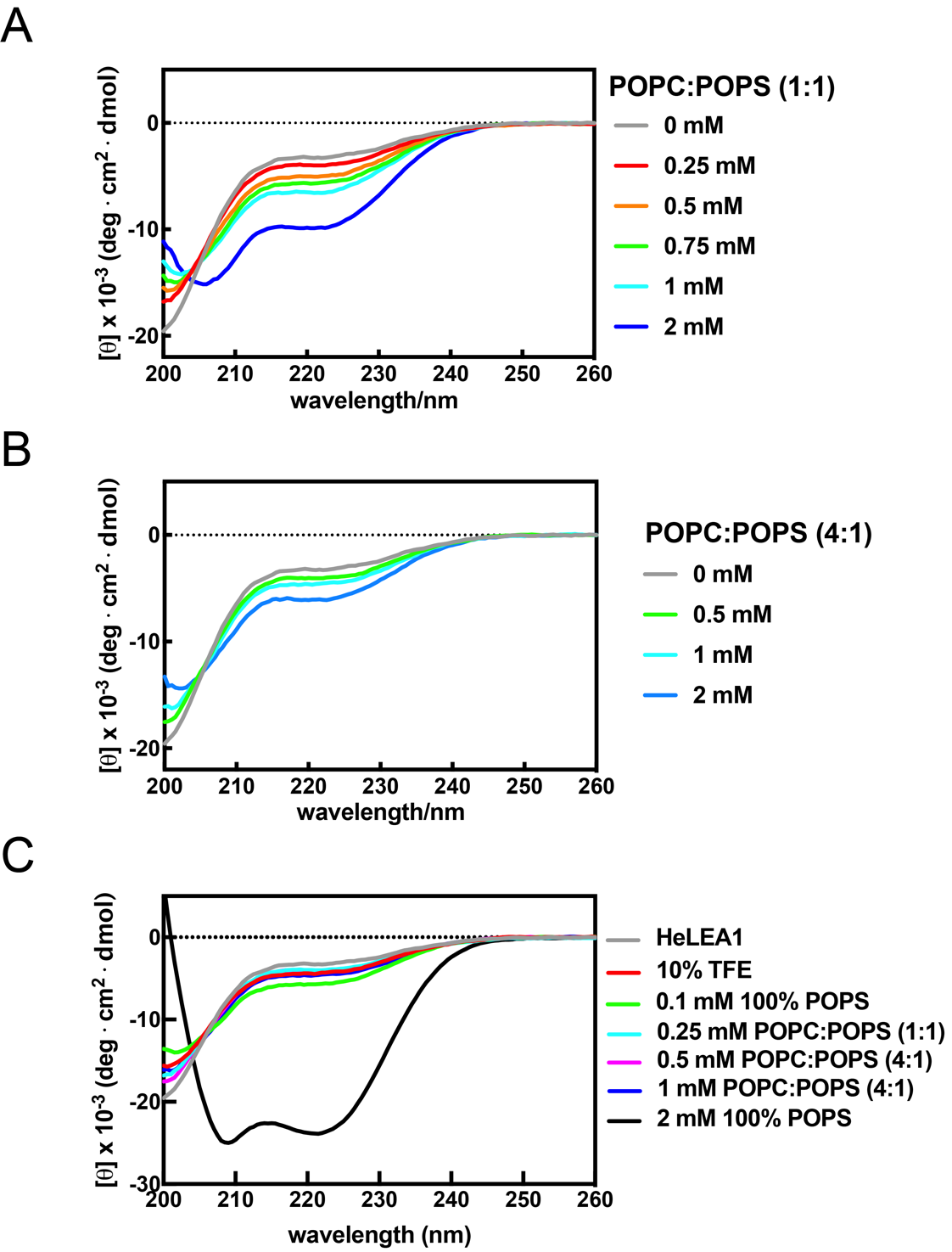


**Fig. S13** **Negatively-charged SUVs trigger disorder-to-helical transition in HeLEA1.** Titration of 10 mM HeLEA1 with increasing concentration of POPC:POPS (1:1) SUVs (**A**) and POPC:POPS (4:1) (**B**). The transition showed a clear isodichroic point at 204nm. **C,** Overlays of HeLEA1 alone (gray), HeLEA1 with 2mM 100% POPS SUVs (black), HeLEA1 with various negatively charged SUVs at low concentrations (green, cyan, magenta, blue), and HeLEA1 with 10% TFE are shown. The data suggest 10% TFE is able to mimic a conformational ensemble that is similar to the early stage of such disorder-to-helical transition induced by negatively-charged SUVs.


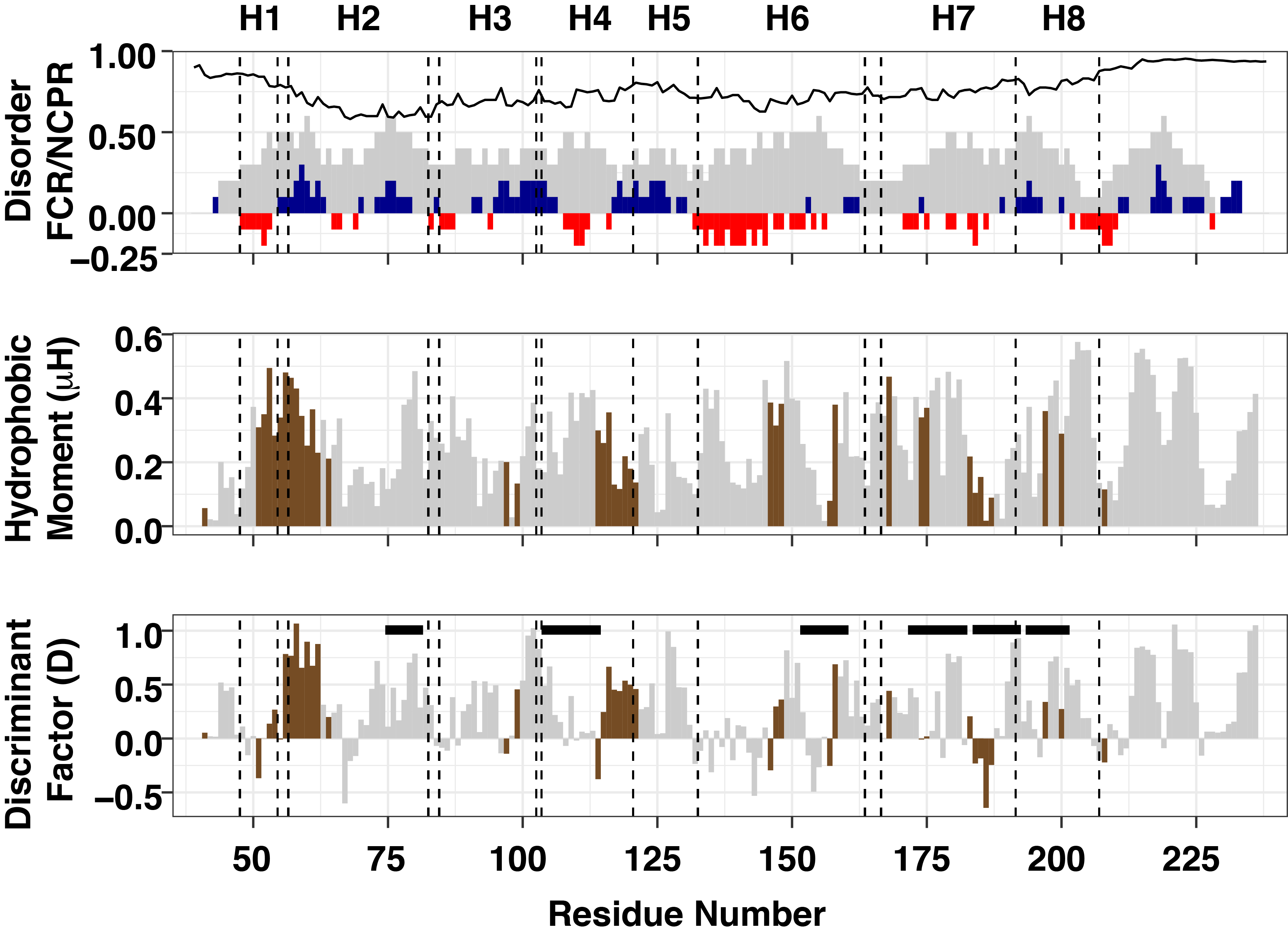


**Fig. S14 Sequence properties of HeLEA1.** **Top**, plot showing IUPred2 predicted disorder propensity (black line), fraction of charged residues (FCR, gray bars), and net charge per residue (NCPR, red/blue bars for net negative/positive charges). **Middle**, distribution of hydrophobic moments of local short amphipathic elements (length of 5). **Bottom**, distribution of lipid binding discriminant factor D of local short amphipathic elements (length of 5), which integrates contribution from hydrophobic moments and charge interactions; black bars indicate conserved LEA motifs mapped in **Fig. 2A**. Regions with increased helical propensity in 10% TFE corresponding to colored residues in **Fig. 2D** are highlighted in brown for hydrophobic moment and lipid binding factor plots.


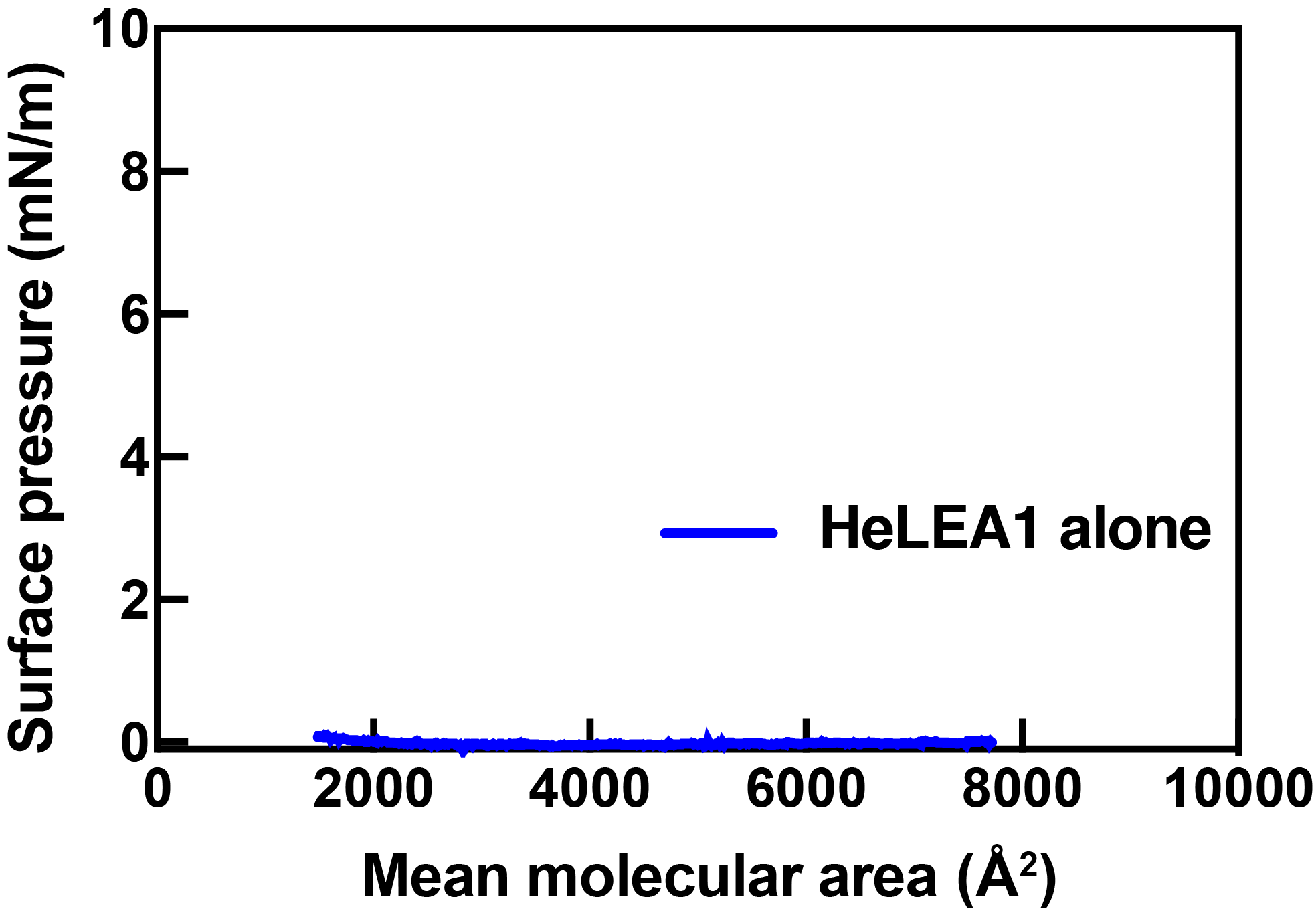


**Fig. S15** Compression isotherm of 3nM HeLEA1 alone, showing negligible mechanical response are air-water interface.


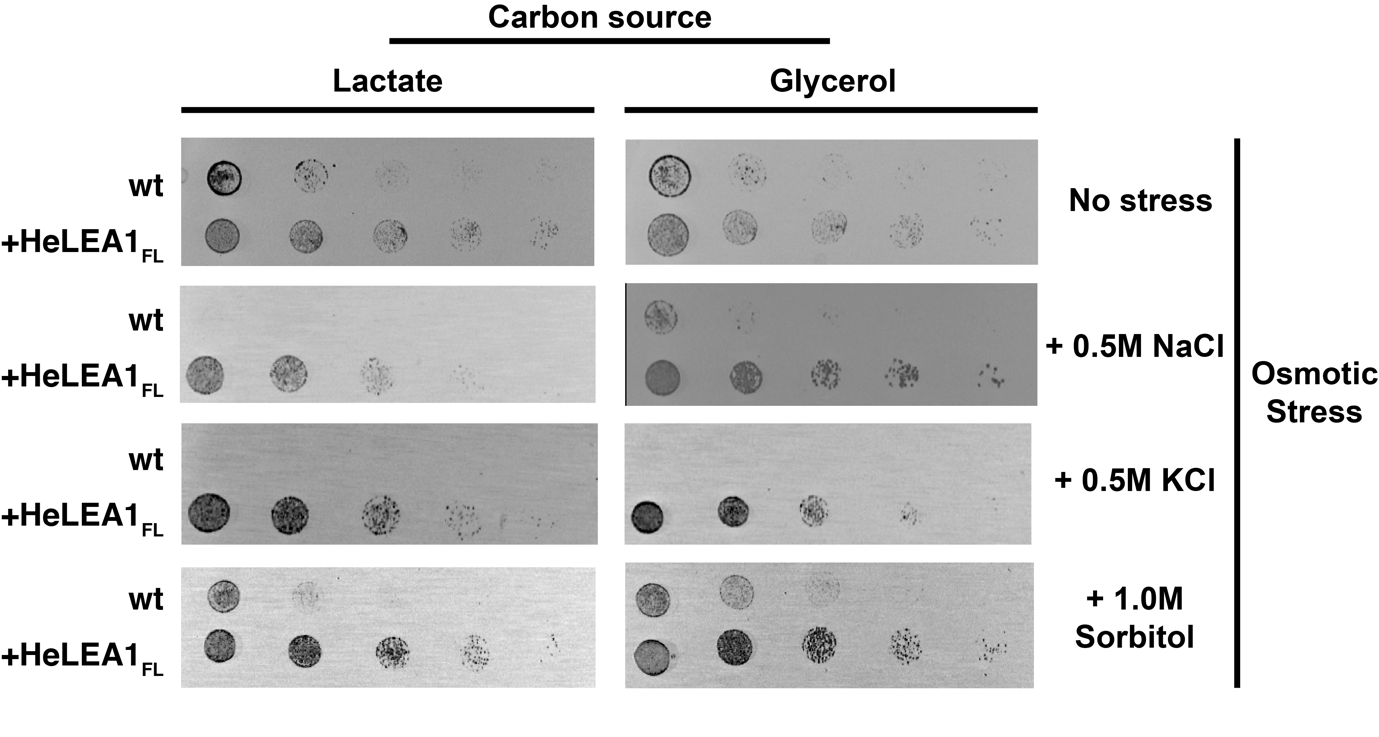


**Fig. S16** HeLEA1_FL_ expression confers tolerance to multiple osmotic stresses on non-fermentable carbon sources. Lactate and glycerol which are both non-fermentable carbon sources were combined with 0.5M NaCl, 0.5M KCl, or 1.0M Sorbitol to mimic varios osmotic stresses. In either case, expression of HeLEA1_FL_ confers a significant growth advantage compared to strains without HeLEA1_FL_ expression at 37 °C.


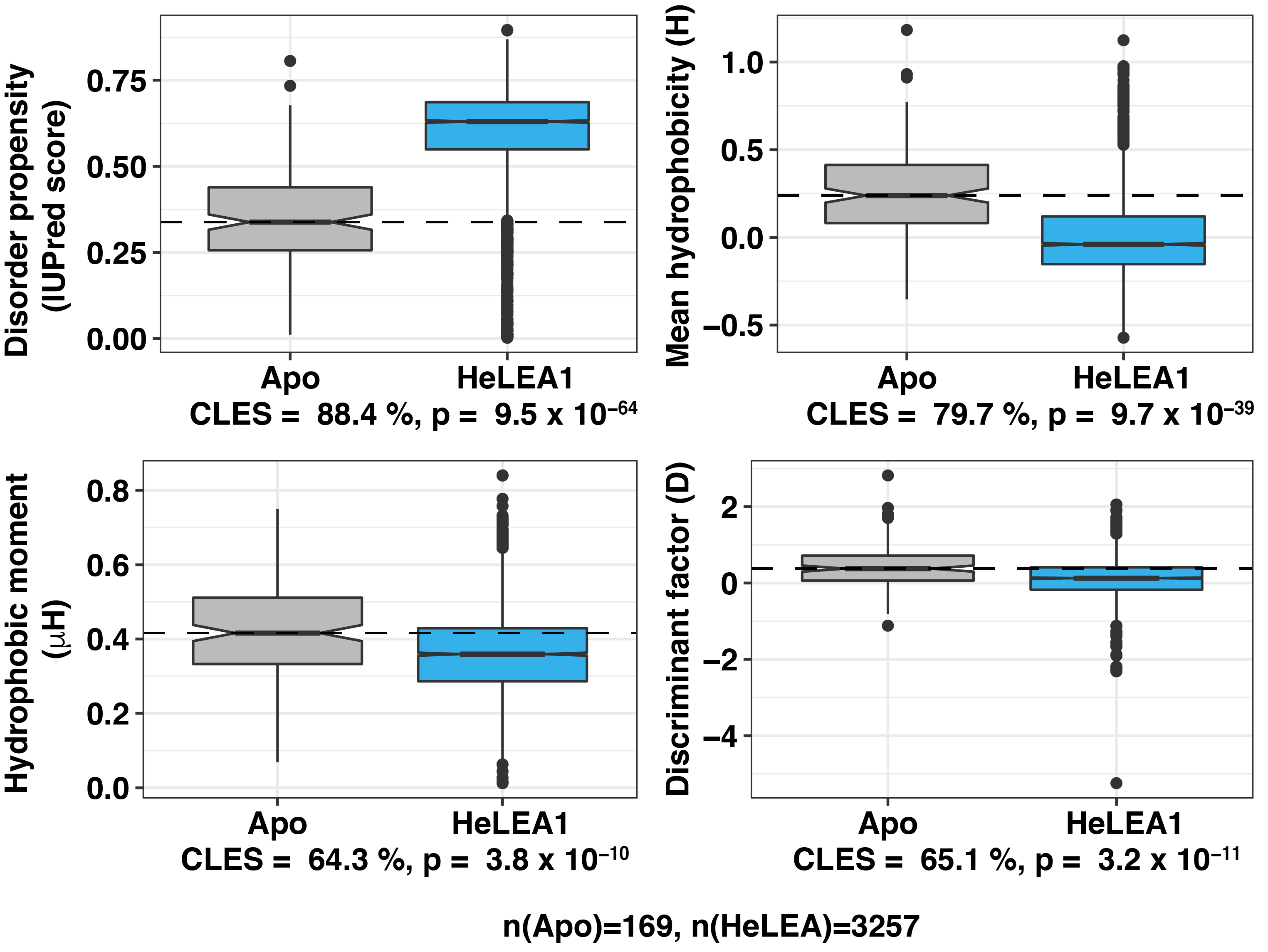


**Fig. S17** HeLEA1 homologs have distinct features that discriminate them from classic lipid-binding helices in apolipoproteins. Amphipathic 3-11 helices in HeLEA1 homologs possess a significantly higher propensity of being intrinsically disordered, less hydrophobic (lower mean hydrophobicity) and amphipathic (lower hydrophobic moment), and has a lower discriminant score D, indicating weaker lipid-binding ability. Each box plot demonstrates specific biophysical parameters of 3-11 amphipathic helices either from human apolipoprotein family (Apo) or HeLEA1 homologs (HeLEA1), using the algorithm described in **Fig. S6**. Each box represents the IQR of the dataset, whiskers represent plus/minus 1.5 IQR from the box hinge, and outliers are plotted as dot.


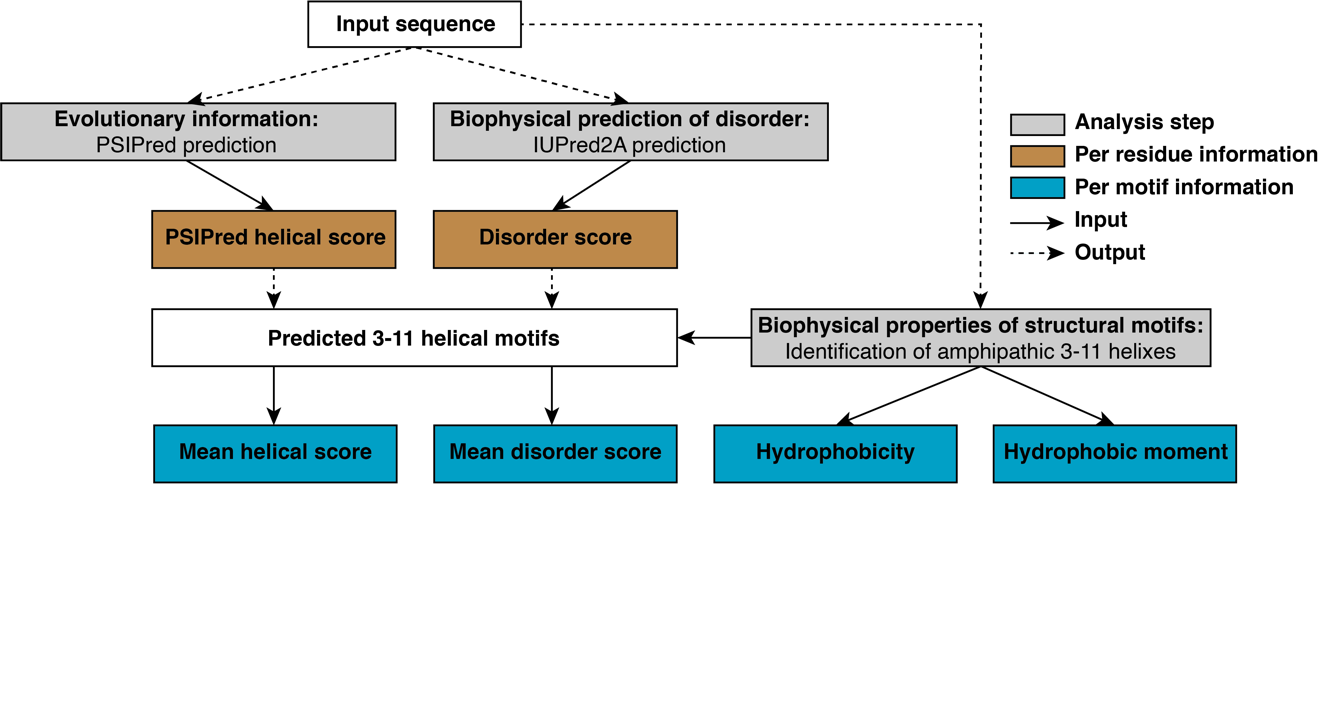


**Fig. S18** Bioinformatics pipeline for discovery of 3-11 helical motifs in HeLEA homologs and sequence properties analyzed for each motif.

**Table S1 Tardigrade-specific homologs (48 sequences from three tardigrade species).**

| **UniProt ID** | **Description** | **Phylum** | **Family** | **Organism** |
| --- | --- | --- | --- | --- |
| **P0CU52** | Cytosolic-abundant heat soluble protein 106094 | Tardigrada | Macrobiotidae | *Paramacrobiotus richtersi* |
| **P0CU51** | Cytosolic-abundant heat soluble protein 107838 | Tardigrada | Macrobiotidae | *Paramacrobiotus richtersi* |
| **P0CU43** | Cytosolic-abundant heat soluble protein 77580 | Tardigrada | Hypsibiidae | *Hypsibius exemplaris* |
| **P0CU47** | Cytosolic-abundant heat soluble protein 89226 | Tardigrada | Hypsibiidae | *Hypsibius exemplaris* |
| **P0CU48^*^** | Cytosolic-abundant heat soluble protein 94205 | Tardigrada | Hypsibiidae | *Hypsibius exemplaris* |
| **P0CU50^*^** | Cytosolic-abundant heat soluble protein 94063 | Tardigrada | Hypsibiidae | *Hypsibius exemplaris* |
| **A0A1D1UM97** | CAHS6 | Tardigrada | Ramazzottiidae | *Ramazzottius varieornatus* |
| **A0A1D1UMG9** | CAHS7 | Tardigrada | Ramazzottiidae | *Ramazzottius varieornatus* |
| **A0A1D1UP25** | CAHS4 | Tardigrada | Ramazzottiidae | *Ramazzottius varieornatus* |
| **A0A1D1UPC3** | CAHS5 | Tardigrada | Ramazzottiidae | *Ramazzottius varieornatus* |
| **A0A1D1UQN2** | CAHS8 | Tardigrada | Ramazzottiidae | *Ramazzottius varieornatus* |
| **A0A1D1VA35** | CAHS9 | Tardigrada | Ramazzottiidae | *Ramazzottius varieornatus* |
| **A0A1D1VKN4** | Uncharacterized protein | Tardigrada | Ramazzottiidae | *Ramazzottius varieornatus* |
| **A0A1D1VMX5** | CAHS11 | Tardigrada | Ramazzottiidae | *Ramazzottius varieornatus* |
| **A0A1D1VNN8** | CAHS12 | Tardigrada | Ramazzottiidae | *Ramazzottius varieornatus* |
| **A0A1D1VRJ6** | CAHS13 | Tardigrada | Ramazzottiidae | *Ramazzottius varieornatus* |
| **A0A1D1VS35** | CAHS10 | Tardigrada | Ramazzottiidae | *Ramazzottius varieornatus* |
| **A0A1D1VVD2** | CAHS13 | Tardigrada | Ramazzottiidae | *Ramazzottius varieornatus* |
| **A0A1D1VXT0** | Uncharacterized protein | Tardigrada | Ramazzottiidae | *Ramazzottius varieornatus* |
| **A0A1D1VYP2** | Uncharacterized protein | Tardigrada | Ramazzottiidae | *Ramazzottius varieornatus* |
| **A0A1D1VZY1** | CAHS14 | Tardigrada | Ramazzottiidae | *Ramazzottius varieornatus* |
| **A0A1D1W0P6** | Uncharacterized protein | Tardigrada | Ramazzottiidae | *Ramazzottius varieornatus* |
| **A0A1D1W0X5** | CAHS15 | Tardigrada | Ramazzottiidae | *Ramazzottius varieornatus* |
| **A0A1D1W6J1** | CAHS16 | Tardigrada | Ramazzottiidae | *Ramazzottius varieornatus* |
| **A0A1W0WE44** | Uncharacterized protein | Tardigrada | Hypsibiidae | *Hypsibius exemplaris* |
| **A0A1W0WK44** | Uncharacterized protein | Tardigrada | Hypsibiidae | *Hypsibius exemplaris* |
| **A0A1W0WK50** | Uncharacterized protein | Tardigrada | Hypsibiidae | *Hypsibius exemplaris* |
| **A0A1W0WK96** | Uncharacterized protein | Tardigrada | Hypsibiidae | *Hypsibius exemplaris* |
| **A0A1W0WNR6** | Uncharacterized protein | Tardigrada | Hypsibiidae | *Hypsibius exemplaris* |
| **A0A1W0WNS8** | Uncharacterized protein | Tardigrada | Hypsibiidae | *Hypsibius exemplaris* |
| **A0A1W0WNT0** | Uncharacterized protein | Tardigrada | Hypsibiidae | *Hypsibius exemplaris* |
| **A0A1W0WNY6** | Uncharacterized protein | Tardigrada | Hypsibiidae | *Hypsibius exemplaris* |
| **A0A1W0WT61** | Uncharacterized protein | Tardigrada | Hypsibiidae | *Hypsibius exemplaris* |
| **A0A1W0WYK1** | Uncharacterized protein | Tardigrada | Hypsibiidae | *Hypsibius exemplaris* |
| **A0A1W0X1Q9** | Uncharacterized protein | Tardigrada | Hypsibiidae | *Hypsibius exemplaris* |
| **A0A1W0X224** | Uncharacterized protein | Tardigrada | Hypsibiidae | *Hypsibius exemplaris* |
| **A0A1W0X234** | Uncharacterized protein | Tardigrada | Hypsibiidae | *Hypsibius exemplaris* |
| **A0A1W0X248** | Uncharacterized protein | Tardigrada | Hypsibiidae | *Hypsibius exemplaris* |
| **A0A1W0X2E6** | Uncharacterized protein | Tardigrada | Hypsibiidae | *Hypsibius exemplaris* |
| **A0A1W0X2I1** | Uncharacterized protein | Tardigrada | Hypsibiidae | *Hypsibius exemplaris* |
| **A0A1W0X2T1** | Uncharacterized protein | Tardigrada | Hypsibiidae | *Hypsibius exemplaris* |
| **A0A1W0X5J2** | Uncharacterized protein | Tardigrada | Hypsibiidae | *Hypsibius exemplaris* |
| **A0A1W0X742** | Uncharacterized protein | Tardigrada | Hypsibiidae | *Hypsibius exemplaris* |
| **J7M3T1** | Cytosolic-abundant heat soluble protein 3 | Tardigrada | Ramazzottiidae | *Ramazzottius varieornatus* |
| **J7M799** | Cytosolic-abundant heat soluble protein 1 | Tardigrada | Ramazzottiidae | *Ramazzottius varieornatus* |
| **J7MDG6** | Cytosolic-abundant heat soluble protein 2 | Tardigrada | Ramazzottiidae | *Ramazzottius varieornatus* |
| **P0CU44** | Cytosolic-abundant heat soluble protein 77611 | Tardigrada | Hypsibiidae | *Hypsibius exemplaris* |
| **P0CU45** | Cytosolic-abundant heat soluble protein 94205 | Tardigrada | Hypsibiidae | *Hypsibius exemplaris* |
| **P0CU46** | Cytosolic-abundant heat soluble protein 86272 | Tardigrada | Hypsibiidae | *Hypsibius exemplaris* |

*P0CU48 and P0CU50 have identical protein sequences.

**Table S2 HeLEA1 family homologs.***

| **UniProt ID** | **Description** | **(Super)**  **Kingdom** | **Phylum** | **Organism** | **GO Term** | **PFAM** |
| --- | --- | --- | --- | --- | --- | --- |
| **P0CU49** | Cytosolic-abundant heat soluble protein 68135† (HeLEA1) | Metazoa | Tardigrada | *Hypsibius exemplaris* | None | None |
| **A0A0E4AVP3** | Group 3 late-embryogenesis abundant protein\| mitochondrial | Metazoa | Tardigrada | *Ramazzottius varieornatus* | None | None |
| **A0A1I7UWF9** | Uncharacterized protein | Metazoa | Nematoda | *Caenorhabditis tropicalis.* | 0008289 lipid binding | PF01442 |
| **A0A260ZXL8** | Uncharacterized protein | Metazoa | Nematoda | *Caenorhabditis remanei* | 0008289 lipid binding | PF01442 |
| **A0A260ZXQ5** | Uncharacterized protein | Metazoa | Nematoda | *Caenorhabditis remanei* | 0008289 lipid binding | PF01442 |
| **A0A260ZYZ6** | Uncharacterized protein | Metazoa | Nematoda | *Caenorhabditis remanei* | 0008289 lipid binding | PF01442 |
| **A0A260ZZ29** | Uncharacterized protein | Metazoa | Nematoda | *Caenorhabditis remanei* | 0008289 lipid binding | PF01442 |
| **A0A261BJ41** | Uncharacterized protein | Metazoa | Nematoda | *Caenorhabditis latens* | 0008289 lipid binding | PF01442 |
| **A0A2P4V0U1** | Uncharacterized protein | Metazoa | Nematoda | *Caenorhabditis remanei* | 0008289 lipid binding | PF01442 |
| **A0A2P4V0U3** | Uncharacterized protein | Metazoa | Nematoda | *Caenorhabditis remanei* | 0008289 lipid binding | PF01442 |
| **G5EDH9** | Plant late embryo abundant (LEA) related | Metazoa | Nematoda | *Caenorhabditis elegans* | 0008289 lipid binding | PF01442 |
| **G5EEF0** | Plant late embryo abundant (LEA) related | Metazoa | Nematoda | *Caenorhabditis elegans* | 0008289 lipid binding | PF01442 |
| **H2FLK6** | Plant late embryo abundant (LEA) related | Metazoa | Nematoda | *Caenorhabditis elegans* | 0008289 lipid binding | PF01442 |
| **H2FLK7** | Plant late embryo abundant (LEA) related | Metazoa | Nematoda | *Caenorhabditis elegans* | 0008289 lipid binding | PF01442 |
| **H2FLK8** | Plant late embryo abundant (LEA) related | Metazoa | Nematoda | *Caenorhabditis elegans* | 0008289 lipid binding | PF01442 |
| **H2FLK9** | Plant late embryo abundant (LEA) related | Metazoa | Nematoda | *Caenorhabditis elegans* | 0008289 lipid binding | PF01442 |
| **H2FLL0** | Plant late embryo abundant (LEA) related | Metazoa | Nematoda | *Caenorhabditis elegans* | 0008289 lipid binding | PF01442 |
| **H2FLL1** | Plant late embryo abundant (LEA) related | Metazoa | Nematoda | *Caenorhabditis elegans* | 0008289 lipid binding | PF01442 |
| **A0A1H9UYK8** | Uncharacterized protein | Bacteria | Actinobacteria | *Phycicoccus cremeus* | None | PF12277 |
| **A0A1S1RAS2** | Uncharacterized protein | Bacteria | Actinobacteria | *Frankia* sp. NRRL B-16219 | None | PF12277 |
| **A0A1Y3M3B0** | Uncharacterized protein | Bacteria | Actinobacteria | *Arthrobacter agilis* | None | PF12277 |
| **A0A1I7UWI0** | Uncharacterized protein | Metazoa | Nematoda | *Caenorhabditis tropicalis.* | None | PF02987 |
| **A0A2G5TP49** | Uncharacterized protein | Metazoa | Nematoda | *Caenorhabditis nigoni* | None | PF02987 |
| **A0A2G5TP52** | Uncharacterized protein | Metazoa | Nematoda | *Caenorhabditis nigoni* | None | PF02987 |
| **A0A2G5TPR7** | Uncharacterized protein | Metazoa | Nematoda | *Caenorhabditis nigoni.* | None | PF02987 |
| **F5GUH5** | Plant late embryo abundant (LEA) related | Metazoa | Nematoda | *Caenorhabditis elegans* | None | PF02987 |
| **G0NF69** | Uncharacterized protein | Metazoa | Nematoda | *Caenorhabditis brenneri* | None | PF02987 |
| **A0A2G5UAI2** | Uncharacterized protein | Metazoa | Nematoda | *Caenorhabditis nigoni* | None | PF02338 |
| **A0A078J7G7** | BnaCnng36560D protein | Viridiplantae | Streptophyta | *Brassica napus* | None | None |
| **A0A087GJV5** | Uncharacterized protein | Viridiplantae | Streptophyta | *Arabis alpina* | None | None |
| **A0A087GM44** | Uncharacterized protein | Viridiplantae | Streptophyta | *Arabis alpina* | None | None |
| **A0A0D2UQL0** | Uncharacterized protein | Eukaryota | Unclassified | *Capsaspora owczarzaki* (strain ATCC 30864) | None | None |
| **A0A0D2VWU8** | Uncharacterized protein | Eukaryota | Unclassified | *Capsaspora owczarzaki* (strain ATCC 30864) | None | None |
| **A0A0D2VZL6** | Uncharacterized protein | Eukaryota | Unclassified | *Capsaspora owczarzaki* (strain ATCC 30864) | None | None |
| **A0A0D3AU26** | Uncharacterized protein | Viridiplantae | Streptophyta | *Brassica oleracea* var. oleracea. | None | None |
| **A0A0D3E6F9** | Uncharacterized protein | Viridiplantae | Streptophyta | *Brassica oleracea* var. oleracea | None | None |
| **A0A109UGY4** | Uncharacterized protein | Bacteria | Firmicutes | *Erysipelothrix larvae* | None | None |
| **A0A132P350** | Phage tail tape measure protein | Bacteria | Firmicutes | *Enterococcus faecium* | None | None |
| **A0A151JYK0** | Laminin subunit alpha-2 | Metazoa | Arthropoda | *Trachymyrmex septentrionalis* | None | None |
| **A0A176WDA2** | Uncharacterized protein | Viridiplantae | Streptophyta | *Marchantia polymorpha* subsp. ruderalis | None | None |
| **A0A178UQA5** | Uncharacterized protein | Viridiplantae | Streptophyta | *Arabidopsis thaliana* | None | None |
| **A0A1D8P660** | Uncharacterized protein | Bacteria | Bacteroidetes | *Lutibacter* sp. LPB0138 | None | None |
| **A0A1E5UFV5** | CBR-LEA-1 domain protein | Bacteria | Bacteroidetes | *Cloacibacterium normanense* | None | None |
| **A0A1I7RYR8** | Uncharacterized protein | Metazoa | Nematoda | *Bursaphelenchus xylophilus* | None | None |
| **A0A1I7S4Z7** | Uncharacterized protein | Metazoa | Nematoda | *Bursaphelenchus xylophilus* | None | None |
| **A0A1S8KJD9** | Phage tail tape measure protein | Bacteria | Firmicutes | *Enterococcus faecium* | None | None |
| **A0A1Y3N4D1** | Uncharacterized protein | Fungi | Chytridiomycota | *Piromyces* sp. (strain E2) | None | None |
| **A0A1Z5JAE4** | Uncharacterized protein | Eukaryota | Bacillariophyta | *Fistulifera solaris* | None | None |
| **A0A1Z5JG32** | Uncharacterized protein | Eukaryota | Bacillariophyta | *Fistulifera solaris* | None | None |
| **A0A260YM94** | Uncharacterized protein | Metazoa | Nematoda | *Caenorhabditis latens.* | None | None |
| **A0A260ZXU5** | Uncharacterized protein | Metazoa | Nematoda | *Caenorhabditis remanei* | None | None |
| **A0A261C8V8** | Uncharacterized protein | Metazoa | Nematoda | *Caenorhabditis latens* | None | None |
| **A0A2G5TP72** | Uncharacterized protein | Metazoa | Nematoda | *Caenorhabditis nigoni* | None | None |
| **A0A2G5TPM5** | Uncharacterized protein | Metazoa | Nematoda | *Caenorhabditis nigoni* | None | None |
| **A0A2H6HFT2** | Uncharacterized protein | Bacteria | Unclassified | bacterium BMS3Bbin02 | None | None |
| **A0A2N3GFW3** | Uncharacterized protein | Bacteria | Actinobacteria | *Actinobacteria* bacterium HGW-Actinobacteria-2 | None | None |
| **A0A2P4V0V3** | Uncharacterized protein | Metazoa | Nematoda | *Caenorhabditis remanei* | None | None |
| **A0A2R6W6L8** | Uncharacterized protein | Viridiplantae | Streptophyta | *Marchantia polymorpha* | None | None |
| **A0A2R6WAA3** | Uncharacterized protein | Viridiplantae | Streptophyta | *Marchantia polymorpha* | None | None |
| **A0A2R6WAB3** | Uncharacterized protein | Viridiplantae | Streptophyta | *Marchantia polymorpha* | None | None |
| **A0A2R6WAD9** | Uncharacterized protein | Viridiplantae | Streptophyta | *Marchantia polymorpha* | None | None |
| **A0A2R6XB50** | Uncharacterized protein | Viridiplantae | Streptophyta | *Marchantia polymorpha* | None | None |
| **A0A2R7X3D6** | Uncharacterized protein | Metazoa | Arthropoda | *Oncopeltus fasciatus* | None | None |
| **A0A2S7I8S5** | Uncharacterized protein | Bacteria | Bacteroidetes | *Cloacibacterium normanense* | None | None |
| **A0A2W5ESR9** | Uncharacterized protein | Bacteria | Bacteroidetes | *Pseudopedobacter saltans* | None | None |
| **A0A2Y9BD83** | Uncharacterized protein | Bacteria | Firmicutes | *Faecalicatena orotica* | None | None |
| **A8DNR5** | LEA-1A | Metazoa | Rotifera | *Adineta ricciae* | None | None |
| **A8WYH4** | Protein CBG04792 | Metazoa | Nematoda | *Caenorhabditis briggsae* | None | None |
| **A8WYJ1** | Protein CBR-LEA-1 | Metazoa | Nematoda | *Caenorhabditis briggsae* | None | None |
| **A8WYK4** | Protein CBG04827 | Metazoa | Nematoda | *Caenorhabditis briggsae* | None | None |
| **B1PM75 **** | **Late-embryogenesis abundant group 3 protein** | **Metazoa** | **Arthropoda** | ***Artemia franciscana*** | **None** | **None** |
| **B7G6G1** | Predicted protein | Eukaryota | Bacillariophyta | *Phaeodactylum tricornutum* (strain CCAP 1055/1) | None | None |
| **D7MFG0** | Late embryogenesis abundant domain-containing protein | Viridiplantae | Streptophyta | *Arabidopsis lyrata* subsp. lyrata | None | None |
| **D7MMJ6** | Late embryogenesis abundant domain-containing protein | Viridiplantae | Streptophyta | *Arabidopsis lyrata* subsp. lyrata | None | None |
| **E3LIS8** | Uncharacterized protein | Metazoa | Nematoda | *Caenorhabditis remanei* | None | None |
| **E3LIT3** | **CRE-LEA-1 protein** | **Metazoa** | **Nematoda** | ***Caenorhabditis remanei*** | **None** | **None** |
| **E3LIT5** | **CRE-LEA-1 protein** | **Metazoa** | **Nematoda** | ***Caenorhabditis remanei*** | **None** | **None** |
| **F0YUN7** | Late embryogenesis abundant protein 76 (LEA 76) | Bacteria | Firmicutes | *Clostridium* sp. D5 | None | None |
| **G0NF78** | Uncharacterized protein | Metazoa | Nematoda | *Caenorhabditis brenneri* | None | None |
| **G0PIE3** | Uncharacterized protein | Metazoa | Nematoda | *Caenorhabditis brenneri* | None | None |
| **G5EFU3** | **Ce-LEA** | **Metazoa** | **Nematoda** | ***Caenorhabditis elegans*** | **None** | **None** |
| **G7ISC0** | **Late embryogenesis abundant domain protein\| putative** | **Viridiplantae** | **Streptophyta** | ***Medicago truncatula*** | **None** | **None** |
| **H2FLK3** | Plant late embryo abundant (LEA) related | Metazoa | Nematoda | *Caenorhabditis elegans* | None | None |
| **H2FLK4** | Plant late embryo abundant (LEA) related | Metazoa | Nematoda | *Caenorhabditis elegans* | None | None |
| **H2FLK5** | Plant late embryo abundant (LEA) related | Metazoa | Nematoda | *Caenorhabditis elegans* | None | None |
| **J6XTJ0** | Tape measure domain protein | Bacteria | Firmicutes | *Enterococcus faecium R496* | None | None |
| **K7GW28** | Uncharacterized protein | Metazoa | Nematoda | *Caenorhabditis japonica* | None | None |
| **K7GW29** | Uncharacterized protein | Metazoa | Nematoda | *Caenorhabditis japonica* | None | None |
| **K7GW30** | Uncharacterized protein | Metazoa | Nematoda | *Caenorhabditis japonica* | None | None |
| **Q1XI24** | **PvLEA3 protein** | **Metazoa** | **Arthropoda** | ***Polypedilum vanderplanki*** | **None** | **None** |
| **Q3E8H9** | **Late embryogenesis abundant protein (LEA) family protein** | **Viridiplantae** | **Streptophyta** | ***Arabidopsis thaliana*** | **None** | **None** |
| **Q6NMC2** | **At5g44310** | **Viridiplantae** | **Streptophyta** | ***Arabidopsis thaliana*** | **None** | **None** |
| **Q8SVY9** | Uncharacterized protein ECU03_1610 | Fungi | Microsporidia | *Encephalitozoon cuniculi* (strain GB-M1) | None | None |
| **Q9FKV7** | **Late embryogenesis abundant protein (LEA) family protein** | **Viridiplantae** | **Streptophyta** | ***Arabidopsis thaliana*** | **None** | **None** |
| **R0GJD5** | Uncharacterized protein | Viridiplantae | Streptophyta | *Capsella rubella* | None | None |
| **V4LXF4** | Uncharacterized protein | Viridiplantae | Streptophyta | *Eutrema salsugineum* | None | None |

*We identified 96 proteins from 44 species, including two tardigrade species

** Homologs that has a record in LEAPdb are highlighted by bold underline.

†Annotation for HeLEA1 from UniProt.

**Table S3** **Summary of estimated radius of gyration (R_g_) for HeLEA1 in solution.**

| **Entry** | **SAXSonIDPs fit*** | **ASTEROIDS selection†** | **Random coil control†** |
| --- | --- | --- | --- |
| 0% TFE | 58.8 ± 0.2 Å | 55 ± 9 Å | 44 ± 9 Å |
| 10% TFE | 46.3 ± 0.3 Å | 50 ± 10 Å | 42 ± 9 Å |

*The error reported represents error of the fitting.

†The error reported represents the heterogeneity of the ensemble, calculated by the standard deviation of the R_g_ of the ensemble.

**Table S4 Localization prediction of HeLEA1 family homologs.**

| **UniProt ID** | **Description** | **Kingdom** | **Phylum** | **Organism** | **TargetP prediction*** | **PSORT prediction** | **Match** |
| --- | --- | --- | --- | --- | --- | --- | --- |
| **A0A0E4AVP3** | Group 3 late-embryogenesis abundant protein\| mitochondrial | Metazoa | Tardigrada | *Ramazzottius varieornatus* | mTP | Mitochondrial | Y |
| **P0CU49** | Cytosolic-abundant heat soluble protein 68135 | Metazoa | Tardigrada | *Hypsibius dujardini* | mTP | Mitochondrial | Y |
| **A0A1I7UWF9** | Uncharacterized protein | Metazoa | Nematoda | *Caenorhabditis tropicalis* | OTHER | Nucleic | Y |
| **A0A260ZXL8** | Uncharacterized protein | Metazoa | Nematoda | *Caenorhabditis remanei* | OTHER | Nucleic | Y |
| **A0A260ZXQ5** | Uncharacterized protein | Metazoa | Nematoda | *Caenorhabditis remanei* | OTHER | Nucleic | Y |
| **A0A260ZYZ6** | Uncharacterized protein | Metazoa | Nematoda | *Caenorhabditis remanei* | OTHER | Nucleic | Y |
| **A0A260ZZ29** | Uncharacterized protein | Metazoa | Nematoda | *Caenorhabditis remanei* | OTHER | Nucleic | Y |
| **A0A261BJ41** | Uncharacterized protein | Metazoa | Nematoda | *Caenorhabditis latens* | OTHER | Nucleic | Y |
| **A0A2P4V0U1** | Uncharacterized protein | Metazoa | Nematoda | *Caenorhabditis remanei* | OTHER | Nucleic | Y |
| **A0A2P4V0U3** | Uncharacterized protein | Metazoa | Nematoda | *Caenorhabditis remanei* | OTHER | Nucleic | Y |
| **G5EDH9** | Plant late embryo abundant (LEA) related | Metazoa | Nematoda | *Caenorhabditis elegans* | OTHER | Nucleic | Y |
| **G5EEF0** | Plant late embryo abundant (LEA) related | Metazoa | Nematoda | *Caenorhabditis elegans* | OTHER | Nucleic | Y |
| **H2FLK6** | Plant late embryo abundant (LEA) related | Metazoa | Nematoda | *Caenorhabditis elegans* | OTHER | Nucleic | Y |
| **H2FLK7** | Plant late embryo abundant (LEA) related | Metazoa | Nematoda | *Caenorhabditis elegans* | OTHER | Nucleic | Y |
| **H2FLK8** | Plant late embryo abundant (LEA) related | Metazoa | Nematoda | *Caenorhabditis elegans* | OTHER | Nucleic | Y |
| **H2FLK9** | Plant late embryo abundant (LEA) related | Metazoa | Nematoda | *Caenorhabditis elegans* | OTHER | Nucleic | Y |
| **H2FLL0** | Plant late embryo abundant (LEA) related | Metazoa | Nematoda | *Caenorhabditis elegans* | OTHER | Nucleic | Y |
| **H2FLL1** | Plant late embryo abundant (LEA) related | Metazoa | Nematoda | *Caenorhabditis elegans* | OTHER | Nucleic | Y |
| **A0A1H9UYK8** | Uncharacterized protein | NA | Actinobacteria | *Phycicoccus cremeus* | OTHER | Cytoplasmic | Y |
| **A0A1S1RAS2** | Uncharacterized protein | NA | Actinobacteria | *Frankia* sp. NRRL B-16219 | OTHER | Cytoplasmic | Y |
| **A0A1Y3M3B0** | Uncharacterized protein | NA | Actinobacteria | *Arthrobacter agilis* | OTHER | Cytoplasmic | Y |
| **A0A1I7UWI0** | Uncharacterized protein | Metazoa | Nematoda | *Caenorhabditis tropicalis* | SP | Extracellular | Y |
| **A0A2G5TP49** | Uncharacterized protein | Metazoa | Nematoda | *Caenorhabditis nigoni* | OTHER | Mitochondrial | N |
| **A0A2G5TP52** | Uncharacterized protein | Metazoa | Nematoda | *Caenorhabditis nigoni* | OTHER | Mitochondrial | N |
| **A0A2G5TPR7** | Uncharacterized protein | Metazoa | Nematoda | *Caenorhabditis nigoni* | OTHER | Mitochondrial | N |
| **F5GUH5** | Plant late embryo abundant (LEA) related | Metazoa | Nematoda | *Caenorhabditis elegans* | OTHER | Cytosolic | Y |
| **G0NF69** | Uncharacterized protein | Metazoa | Nematoda | *Caenorhabditis brenneri* | SP | Extracellular | Y |
| **A0A2G5UAI2** | Uncharacterized protein | Metazoa | Nematoda | *Caenorhabditis nigoni* | OTHER | Nucleic | Y |
| **A0A078J7G7** | BnaCnng36560D protein | Viridiplantae | Streptophyta | *Brassica napus* | mTP | Chloroplast | N |
| **A0A087GJV5** | Uncharacterized protein | Viridiplantae | Streptophyta | *Arabis alpina* | cTP | Chloroplast | Y |
| **A0A087GM44** | Uncharacterized protein | Viridiplantae | Streptophyta | *Arabis alpina* | cTP | Chloroplast | Y |
| **A0A0D2UQL0** | Uncharacterized protein | NA | NA | *Capsaspora owczarzaki* (strain ATCC 30864) | SP | Mitochondrial | N |
| **A0A0D2VWU8** | Uncharacterized protein | NA | NA | *Capsaspora owczarzaki* (strain ATCC 30864) | OTHER | Nucleic | Y |
| **A0A0D2VZL6** | Uncharacterized protein | NA | NA | *Capsaspora owczarzaki* (strain ATCC 30864) | SP | Mitochondrial | N |
| **A0A0D3AU26** | Uncharacterized protein | Viridiplantae | Streptophyta | *Brassica oleracea* var. oleracea | cTP | Chloroplast | Y |
| **A0A0D3E6F9** | Uncharacterized protein | Viridiplantae | Streptophyta | *Brassica oleracea* var. oleracea | mTP | Chloroplast | N |
| **A0A109UGY4** | Uncharacterized protein | NA | Firmicutes | *Erysipelothrix larvae* | OTHER | Cytoplasmic | Y |
| **A0A132P350** | Phage tail tape measure protein | NA | Firmicutes | *Enterococcus faecium* | OTHER | Plasma Membrane | Y |
| **A0A151JYK0** | Laminin subunit alpha-2 | Metazoa | Arthropoda | *Trachymyrmex septentrionalis* | SP | ER | Y |
| **A0A176WDA2** | Uncharacterized protein | Viridiplantae | Streptophyta | *Marchantia polymorpha* subsp. ruderalis | OTHER | Nucleic | Y |
| **A0A178UQA5** | Uncharacterized protein | Viridiplantae | Streptophyta | *Arabidopsis thaliana* | cTP | Chloroplast | Y |
| **A0A1D8P660** | Uncharacterized protein | NA | Bacteroidetes | *Lutibacter* sp. LPB0138 | OTHER | Cytoplasmic | Y |
| **A0A1E5UFV5** | CBR-LEA-1 domain protein | NA | Bacteroidetes | *Cloacibacterium normanense* | OTHER | Cytoplasmic | Y |
| **A0A1I7RYR8** | Uncharacterized protein | Metazoa | Nematoda | *Bursaphelenchus xylophilus* | SP | Extracellular | Y |
| **A0A1I7S4Z7** | Uncharacterized protein | Metazoa | Nematoda | *Bursaphelenchus xylophilus* | SP | Extracellular | Y |
| **A0A1S8KJD9** | Phage tail tape measure protein | NA | Firmicutes | *Enterococcus faecium* | OTHER | Plasma Membrane | Y |
| **A0A1Y3N4D1** | Uncharacterized protein | Fungi | Chytridiomycota | *Piromyces* sp. (strain E2) | SP | Extracellular | Y |
| **A0A1Z5JAE4** | Uncharacterized protein | NA | Bacillariophyta | *Fistulifera solaris* | SP | Chloroplast | N |
| **A0A1Z5JG32** | Uncharacterized protein | NA | Bacillariophyta | *Fistulifera solaris* | SP | Chloroplast | N |
| **A0A260YM94** | Uncharacterized protein | Metazoa | Nematoda | *Caenorhabditis latens* | OTHER | Cytosolic | Y |
| **A0A260ZXU5** | Uncharacterized protein | Metazoa | Nematoda | *Caenorhabditis remanei* | SP | Extracellular | Y |
| **A0A261C8V8** | Uncharacterized protein | Metazoa | Nematoda | *Caenorhabditis latens* | SP | Extracellular | Y |
| **A0A2G5TP72** | Uncharacterized protein | Metazoa | Nematoda | *Caenorhabditis nigoni* | OTHER | Cytosolic | Y |
| **A0A2G5TPM5** | Uncharacterized protein | Metazoa | Nematoda | *Caenorhabditis nigoni* | SP | Extracellular | Y |
| **A0A2H6HFT2** | Uncharacterized protein | NA | NA | bacterium BMS3Bbin02 | OTHER | Cytoplasmic | Y |
| **A0A2N3GFW3** | Uncharacterized protein | NA | Actinobacteria | *Actinobacteria* bacterium HGW-Actinobacteria-2 | OTHER | Cytoplasmic | Y |
| **A0A2P4V0V3** | Uncharacterized protein | Metazoa | Nematoda | *Caenorhabditis remanei* | SP | Extracellular | Y |
| **A0A2R6W6L8** | Uncharacterized protein | Viridiplantae | Streptophyta | *Marchantia polymorpha* | OTHER | Nucleic | Y |
| **A0A2R6WAA3** | Uncharacterized protein | Viridiplantae | Streptophyta | *Marchantia polymorpha* | OTHER | Nucleic | Y |
| **A0A2R6WAB3** | Uncharacterized protein | Viridiplantae | Streptophyta | *Marchantia polymorpha* | OTHER | Peroxisome | Y |
| **A0A2R6WAD9** | Uncharacterized protein | Viridiplantae | Streptophyta | *Marchantia polymorpha* | OTHER | Nucleic | Y |
| **A0A2R6XB50** | Uncharacterized protein | Viridiplantae | Streptophyta | *Marchantia polymorpha* | cTP | Chloroplast | Y |
| **A0A2R7X3D6** | Uncharacterized protein | Metazoa | Arthropoda | *Oncopeltus fasciatus* | OTHER | Cytosolic | Y |
| **A0A2S7I8S5** | Uncharacterized protein | NA | Bacteroidetes | *Cloacibacterium normanense* | OTHER | Cytoplasmic | Y |
| **A0A2W5ESR9** | Uncharacterized protein | NA | Bacteroidetes | *Pseudopedobacter saltans* | OTHER | Cytoplasmic | Y |
| **A0A2Y9BD83** | Uncharacterized protein | NA | Firmicutes | *Faecalicatena orotica* | OTHER | Cytoplasmic | Y |
| **A8DNR5** | Lea-1A | Metazoa | Rotifera | *Adineta ricciae* | SP | ER | Y |
| **A8WYH4** | Protein CBG04792 | Metazoa | Nematoda | *Caenorhabditis briggsae* | SP | Extracellular | Y |
| **A8WYJ1** | Protein CBR-LEA-1 | Metazoa | Nematoda | *Caenorhabditis briggsae* | OTHER | Cytosolic | Y |
| **A8WYK4** | Protein CBG04827 | Metazoa | Nematoda | *Caenorhabditis briggsae* | OTHER | Cytosolic | Y |
| **B1PM75** | Late-embryogenesis abundant group 3 protein | Metazoa | Arthropoda | *Artemia franciscana* | OTHER | Cytosolic | Y |
| **B7G6G1** | Predicted protein | NA | Bacillariophyta | *Phaeodactylum tricornutum* (strain CCAP 1055/1) | SP | Chloroplast | N |
| **D7MFG0** | Late embryogenesis abundant domain-containing protein | Viridiplantae | Streptophyta | *Arabidopsis lyrata* subsp. lyrata | cTP | Chloroplast | Y |
| **D7MMJ6** | Late embryogenesis abundant domain-containing protein | Viridiplantae | Streptophyta | *Arabidopsis lyrata* subsp. lyrata | cTP | Chloroplast | Y |
| **E3LIS8** | Uncharacterized protein | Metazoa | Nematoda | *Caenorhabditis remanei* | SP | Extracellular | Y |
| **E3LIT3** | CRE-LEA-1 protein | Metazoa | Nematoda | *Caenorhabditis remanei* | OTHER | Cytosolic | Y |
| **E3LIT5** | CRE-LEA-1 protein | Metazoa | Nematoda | *Caenorhabditis remanei* | OTHER | Cytosolic | Y |
| **F0YUN7** | Late embryogenesis abundant protein 76 (LEA 76) | NA | Firmicutes | *Clostridium* sp. D5. | OTHER | Cytoplasmic | Y |
| **G0NF78** | Uncharacterized protein | Metazoa | Nematoda | *Caenorhabditis brenneri* | OTHER | Cytosolic | Y |
| **G0PIE3** | Uncharacterized protein | Metazoa | Nematoda | *Caenorhabditis brenneri* | OTHER | Cytosolic | Y |
| **G5EFU3** | Ce-LEA | Metazoa | Nematoda | *Caenorhabditis elegans* | OTHER | Nucleic | Y |
| **G7ISC0** | Late embryogenesis abundant domain protein\| putative | Viridiplantae | Streptophyta | *Medicago truncatula* | mTP | Chloroplast | N |
| **H2FLK3** | Plant late embryo abundant (LEA) related | Metazoa | Nematoda | *Caenorhabditis elegans* | OTHER | Nucleic | Y |
| **H2FLK4** | Plant late embryo abundant (LEA) related | Metazoa | Nematoda | *Caenorhabditis elegans* | OTHER | Nucleic | Y |
| **H2FLK5** | Plant late embryo abundant (LEA) related | Metazoa | Nematoda | *Caenorhabditis elegans* | OTHER | Nucleic | Y |
| **J6XTJ0** | Tape measure domain protein | NA | Firmicutes | *Enterococcus faecium* R496 | OTHER | Plasma Membrane | Y |
| **K7GW28** | Uncharacterized protein | Metazoa | Nematoda | *Caenorhabditis japonica* | OTHER | Cytosolic | Y |
| **K7GW29** | Uncharacterized protein | Metazoa | Nematoda | *Caenorhabditis japonica* | OTHER | Cytosolic | Y |
| **K7GW30** | Uncharacterized protein | Metazoa | Nematoda | *Caenorhabditis japonica* | OTHER | Cytosolic | Y |
| **Q1XI24** | PvLEA3 protein | Metazoa | Arthropoda | *Polypedilum vanderplanki* | OTHER | ER | N |
| **Q3E8H9** | Late embryogenesis abundant protein (LEA) family protein | Viridiplantae | Streptophyta | *Arabidopsis thaliana* | OTHER | Nucleic | Y |
| **Q6NMC2** | At5g44310 | Viridiplantae | Streptophyta | *Arabidopsis thaliana* | cTP | Chloroplast | Y |
| **Q8SVY9** | Uncharacterized protein ECU03_1610 | Fungi | Microsporidia | *Encephalitozoon cuniculi* (strain GB-M1) | OTHER | Nucleic | Y |
| **Q9FKV7** | Late embryogenesis abundant protein (LEA) family protein | Viridiplantae | Streptophyta | *Arabidopsis thaliana* | cTP | Chloroplast | Y |
| **R0GJD5** | Uncharacterized protein | Viridiplantae | Streptophyta | *Capsella rubella* | cTP | Chloroplast | Y |
| **V4LXF4** | Uncharacterized protein | Viridiplantae | Streptophyta | *Eutrema salsugineum* | cTP | Chloroplast | Y |

* mTP, mitochondrial targeting sequence; cTP, chloroplast targeting sequence; SP, signal peptide; ER, endoplasmic reticulum.

**Table S5 Strains and plasmids used in this work.**

| **Yeast strains** | | |  |
| --- | --- | --- | --- |
| **Name** | **Genotype** | **Use in the study** | **Notes** |
| **YOY249** | MATa his3Δ1 leu2Δ0 met15Δ0 ura3Δ0 TOM20-dRFP::LEU2 | Cell imaging | Kindly given by Yohei Ohashi |
| **XLY001** | MATα his3Δ1 leu2Δ0 lys2Δ0 ura3Δ0 can1::KanMX | Functional assay control (Serial dilution) | ATCC collection |
| **XLY002** | MATa his3Δ1 leu2Δ0 met15Δ0 ura3Δ0 can1::P_TDH3_-HeLEA1 | Functional assays | PCR-mediated homologous recombination using XLp008 as parent vector |
| **XLY119** | MATα his3Δ1 leu2Δ0 lys2Δ0 ura3Δ0 can1::KanMX TOM20-EGFP::HIS3MX6 | Functional assays | PCR-mediated homologous recombination made from XLY001 |
| **XLY120** | MATa his3Δ1 leu2Δ0 met15Δ0 ura3Δ0 can1::P_TDH3_-HeLEA1 TOM20-EGFP::HIS3MX6 | Functional assays | PCR-mediated homologous recombination made from XLY002 |
| **XLY171** | MATa his3Δ1 leu2Δ0 met15Δ0 ura3Δ0 can1::KanMX | Immunoblotting | ATCC collection |
| **Plasmids** | | | |
| **Name** | **Description** | **Use in the study** | **Notes** |
| **XLp008** | pRS416, *TDH3* promoter, full-length HeLEA1 | PCR-mediated integration into yeast genome | Kindly given by Thomas Boothby |
| **XLp022** | pETM11 expression vector, HeLEA1 (39–238) | E. coli expression for protein purification | This truncated HeLEA1 is referred to mature HeLEA1 for structural and biophysical characterizations.  Cloned from XLp008 |
| **XLp023** | pRS416, *TDH3* promoter, full-length HeLEA1, yGFP fusion | Yeast expression and imaging | Cloned from XLp008 |
| **XLp028** | pRS416, *TDH3* promoter, HeLEA1 (39–238), yGFP fusion | Yeast expression and imaging | Cloned from XLp008 |
| **XLp063** | pRS416, *TDH3* promoter, full-length HeLEA1, HA-tagged | Immunoblotting | Cloned from XLp023 |
| **XLp064** | pRS416, *TDH3* promoter, HeLEA1 (39–238), HA-tagged | Immunoblotting | Cloned from XLp028 |
| **XLp080** | pRS416, *TDH3* promoter, MTS (HeLEA1, 1-38), yGFP fusion | Yeast expression and imaging | Cloned from XLp023 |

**SI References**

1. J. A. Riback *et al.*, Innovative scattering analysis shows that hydrophobic disordered proteins are expanded in water. *Science* **358**, 238-241 (2017).
